# Differentiated roles for MreB-actin isologues and autolytic enzymes in *Bacillus subtilis* morphogenesis

**DOI:** 10.1111/mmi.12335

**Published:** 2013-08-04

**Authors:** Patricia Domínguez-Cuevas, Ida Porcelli, Richard A Daniel, Jeff Errington

**Affiliations:** Centre for Bacterial Cell Biology, Newcastle UniversityBaddiley-Clark Building, Richardson Road, Newcastle upon Tyne, NE2 4AX, UK

## Abstract

Cell morphogenesis in most bacteria is governed by spatiotemporal growth regulation of the peptidoglycan cell wall layer. Much is known about peptidoglycan synthesis but regulation of its turnover by hydrolytic enzymes is much less well understood. *Bacillus subtilis* has a multitude of such enzymes. Two of the best characterized are CwlO and LytE: cells lacking both enzymes have a lethal block in cell elongation. Here we show that activity of CwlO is regulated by an ABC transporter, FtsEX, which is required for cell elongation, unlike cell division as in *Escherichia coli*. Actin-like MreB proteins are thought to play a key role in orchestrating cell wall morphogenesis. *B. subtilis* has three MreB isologues with partially differentiated functions. We now show that the three MreB isologues have differential roles in regulation of the CwlO and LytE systems and that autolysins control different aspects of cell morphogenesis. The results add major autolytic activities to the growing list of functions controlled by MreB isologues in bacteria and provide new insights into the different specialized functions of essential cell wall autolysins.

## Introduction

Most bacteria have an external wall that determines cell shape and is crucial for preventing the cell from bursting due to its high internal turgor pressure. The cell wall is also the target for our best antibiotics (particularly β-lactams), and fragments of the wall are recognized by the innate immune system. In virtually all bacteria the cell wall comprises a single huge sac-like molecule of peptidoglycan (PG, also called murein), which is a network of glycan strands cross-linked by peptide bridges. Gram-positive bacteria, such as *Bacillus subtilis*, have a multi-layered cell wall that also contains an additional major class of polymers called teichoic acids, which are anionic in nature and are covalently bound either to the PG (wall teichoic acids; WTA) or to membrane phospholipids (lipoteichoic acids; LTA) (Bhavsar and Brown, 2006; Carballido-Lopez and Formstone, 2007; den Blaauwen *et al*., 2008).

Rod-shaped bacteria enlarge the wall in at least two distinct ways. During growth, the cell elongates along its longitudinal axis, in a process that involves attachment of new glycan strands and cross-linking of peptide side-chains into the pre-existing structure. Elongation alternates with division, in which a plate of new wall material, the division septum, is formed, followed by separation of the daughter cells and maturation of new hemispherical cell poles (den Blaauwen *et al*., 2008; Haeusser and Levin, 2008; Bramkamp and van Baarle, 2009). Insertion of new PG into the wall requires the action of synthases called penicillin-binding proteins (PBPs) (Matsuhashi *et al*., 1990; Goffin and Ghuysen, 1998; Scheffers *et al*., 2004; Scheffers and Pinho, 2005). These enzymes catalyse glycan strand elongation (glycosyl transferase) and/or peptide cross-linking (peptidyl transferase) reactions. PG growth requires the action of hydrolytic enzymes (autolysins) that cleave bonds in the existing PG sacculus to enable surface expansion. These reactions need to be carefully co-ordinated, and PBPs are thought to act in multienzyme complexes, together with autolysins and (in Gram-positives) the enzymes for WTA and possibly LTA synthesis (Holtje, 1996a,b; Kawai *et al*., 2011; Typas *et al*., 2012). However, little is known about the precise composition and organization of these putative complexes.

Most bacteria have distantly related homologues of the two major eukaryotic cytoskeletal proteins: actin and tubulin, called MreB and FtsZ respectively. These proteins are key players in organization of the putative complexes involved in elongation (MreB) and division (FtsZ). The MreB proteins were originally thought to form elongated helical structures that spatially controlled the insertion of new wall material (Jones *et al*., 2001; Daniel and Errington, 2003; Carballido-Lopez and Errington, 2003a; Figge *et al*., 2004; Graumann, 2004; Carballido-Lopez *et al*., 2006; Takacs *et al*., 2010; White *et al*., 2010). Recently, new highly sensitive and higher resolution imaging methods have suggested that MreB proteins form smaller patches or short arcs that move over the cell surface driven by peptidoglycan synthesis (Dominguez-Escobar *et al*., 2011; Garner *et al*., 2011; Reimold *et al*., 2013). Precisely how MreB proteins regulate PG synthesis to achieve cylindrical cell wall elongation in rod-shaped bacteria remains to be resolved.

To complicate matters, *B. subtilis* and many other rod-shaped bacteria have multiple MreB isoforms. *B. subtilis* has three: MreB (in an operon with highly conserved MreC and MreD proteins), Mbl (‘MreB-like’) and MreBH (‘MreB homologue’) (Abhayawardhane and Stewart, 1995; Carballido-Lopez and Errington, 2003a; Defeu Soufo and Graumann, 2004; Carballido-Lopez *et al*., 2006). Mutations in each of the three genes affect cell shape in different ways. Deletion of all three genes is lethal but lethality can be suppressed, generating mutant cells that have a more or less spherical morphology (Schirner and Errington, 2009; Kawai *et al*., 2009b). All three proteins associate with each other and with multiple protein partners that have various functions in cell morphogenesis (Jones *et al*., 2001; Carballido-Lopez *et al*., 2006; Graumann, 2007; Claessen *et al*., 2008; Kawai *et al*., 2009a; 2011; Soufo and Graumann, 2010). It is thought that functional specialization of the three MreB proteins is due at least in part to differences in their spectrum of interacting partners (Carballido-Lopez *et al*., 2006; Kawai *et al*., 2011). Although the molecular details of these specializations remain poorly defined.

In previous work we reported that MreBH interacts with the autolysin LytE, and that this interaction is required to distribute LytE to the cylindrical part of the cell as well as at the division septum (Carballido-Lopez *et al*., 2006). LytE is one of about 35 putative *B. subtilis* autolysins, which can be grouped into 11 families (Smith *et al*., 2000). This multiplicity of genes supports the view that autolytic activity is an important cellular function but analysis of gene function is likely to be complicated by functional overlap or redundancy. Interestingly, Bisicchia *et al*. (2007) discovered that the endopeptidases LytE and CwlO have an overlapping essential role in cell elongation. The genes encoding these enzymes are among a small number of genes that are positively regulated by an essential two-component regulator of cell wall homeostasis, WalKR. The *lytE* and *cwlO* synthetic lethality seems to be caused by a lack of d/l-endopeptidase activity in the lateral cell wall, which in turn blocks cell elongation and provokes cell lysis (Hashimoto *et al*., 2012). While *cwlO* and *lytE* probably contribute to WalKR essentiality, it remains to be determined if they constitute the sole cause (Bisicchia *et al*., 2007).

Autolytic enzymes need to be tightly regulated, although little is presently known about the specific mechanisms involved. Recently, two groups described a novel mechanism of regulation in which ABC-transporter-like complexes regulate the activities of specific endopeptidases (Sham *et al*., 2011; Yang *et al*., 2011). The ABC transporter corresponds to a previously described cell division factor called FtsEX. It seems that the ATPase activity of the nucleotide-binding domain protein (FtsE) provokes a conformational change in the transmembrane component (FtsX) (Yang *et al*., 2012), which in turn activates the PG hydrolytic activity of the cognate autolysins: in *Streptococcus pneumoniae* this is a direct interaction with the putative PcsB autolysin (Sham *et al*., 2011); whereas in *Escherichia coli* activation works through an intermediate periplasmic protein called EnvC, and there are two regulated autolysins, AmiA and AmiB (Yang *et al*., 2011). In both cases activation of the CW hydrolase(s) at the septum is needed to enable the separation of progeny cells after division, explaining at least in part the deleterious effects of inactivation of the FtsEX complex in both microorganisms.

Previous work showed that the *B. subtilis ftsEX* genes are not essential for growth and pointed towards a role in regulation of the initiation of sporulation (Garti-Levi *et al*., 2008). Here we show that FtsEX regulates the activity of one of the major autolysins required for cell elongation in *B. subtilis*, CwlO, and that the mechanism of regulation is similar to that described for FtsEX proteins in other systems. We also demonstrate that FtsEX/CwlO function is controlled by the Mbl homologue. Differential regulation of LytE vs CwlO explains at least in part the different functional specializations of the MreB isologues. Furthermore, the different phenotypic effects arising from deletions in the *lytE* or *cwlO* genes suggest that these endopeptidases have differentiated roles in cell elongation and provide new insights into the control of cell morphogenesis. Another article in this issue (Meisner *et al*., 2013) describes an independent investigation that yielded results similar and complementary to those described here.

## Results

### *ftsE/X* deletions affect cell elongation rather than division in *B. subtilis*

**Fig. 1:**
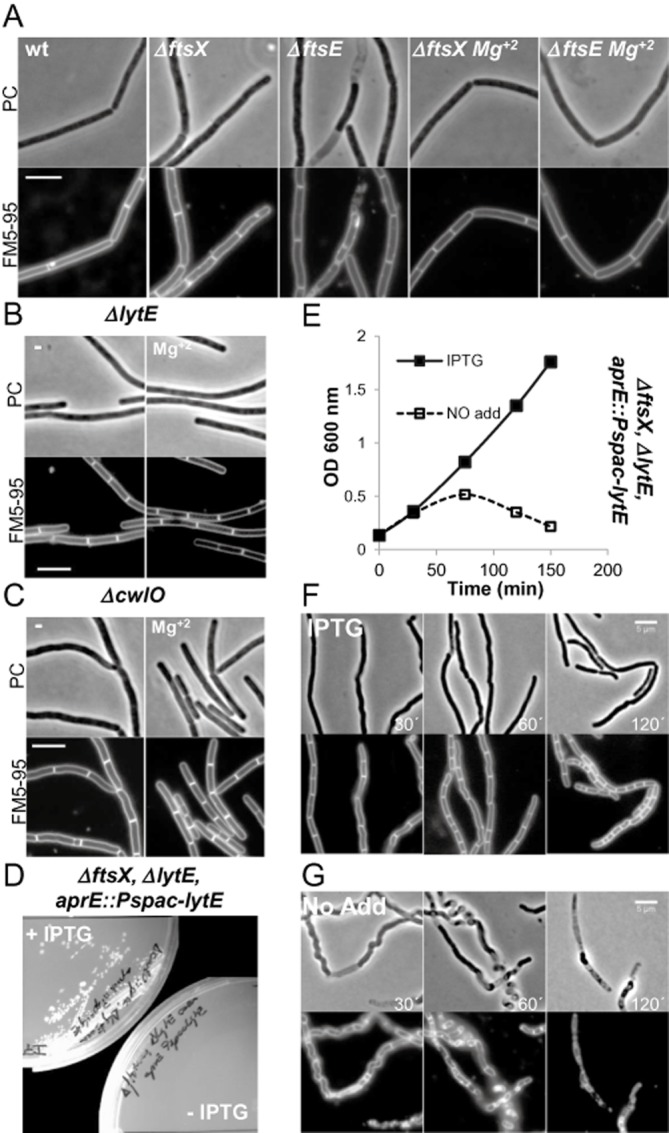
FtsEX mutants are similar to Δ*cwlO* and synthetic lethal with Δ*lytE*. A. Cell morphologies of typical fields of wild-type *B. subtilis* strain 168, Δ*ftsX* *(4501)* and Δ*ftsE* *(4503)* mutant strains growing in a NA plates or in NA plates with supplement of 20 mM Mg^2+^, as indicated. Scale bar represents 5 μm. B and C. Cell morphologies of typical fields of strains PDC464 (Δ*lytE*::*cat*) and PDC463 (Δ*cwlO::spec*) cultured on NA plates in the presence or absence of Mg^2+^ as indicated. Scale bar represents 5 μm. D. Growth of strain PDC492 (Δ*ftsX::neo* Δ*lytE::cat aprE::P_spac_-LytE*) on NA plates with or without 0.5 mM IPTG. E. Growth of strain PDC492 on LB liquid medium in the presence or absence of IPTG. Growth curves (IPTG 0.5 mM, closed symbols; no addition, open symbols). F and G. Effect of LytE depletion on cell morphology. Phase-contrast micrographs and the corresponding membrane staining images were taken at the indicated times during the growth curves in (E). (F) 0.5 mM IPTG added; (G) no IPTG addition. Scale bar represents 5 μm.

Garti-Levi *et al*. (2008) previously showed that *ftsEX* mutants of *B. subtilis* are impaired in the initiation of sporulation. They also noted that, unlike the equivalent mutants of *E. coli*, *B. subtilis ftsEX* mutants are not significantly affected in cell division. Instead, the cells are slightly shorter and wider. We constructed various *ftsE* and *ftsX* mutants and examined their cell phenotype. As reported previously the mutants were indeed wider (cell diameter increased about 23%) and shorter (length reduced about 12%) (Table 1). Under some growth conditions, the normal cylindrical morphology was perturbed, with many cells having a twisted or undulating curved appearance (Fig. 1A). All of these morphological abnormalities were rescued by addition of 20 mM Mg^2+^ to the medium (Fig. 1A, right panels); a phenotype often observed in mutants with defective peptidoglycan synthesis in the lateral CW (Popham and Setlow, 1995; Murray *et al*., 1998; Formstone and Errington, 2005; Leaver and Errington, 2005; Carballido-Lopez *et al*., 2006). In addition to these specific morphological effects, the overall growth rate of *ftsEX* mutant cultures was reduced, especially in low Mg^2+^ medium (Fig. S1A). These results suggest that the main role of *ftsEX* lies in some aspect of cell envelope elongation during vegetative growth.

**Table 1:** Cell length and width measurements

Strain	Genotype	Average cell length (μm)^a^	% wt	Average cell width (μm)^a^	% wt
168	Wt	3.6 ± 0.77	–	0.95 ± 0.081	–
4501	Δ*ftsX*	3.2 ± 0.68	−12	1.17 ± 0.085	+23
4503	Δ*ftsE*	3.2 ± 0.63	−10	1.18 ± 0.084	+24
PDC463	Δ*cwlO*	3.2 ± 0.69	−11	1.18 ± 0.088	+24
PDC464	Δ*lytE*	4.4 ± 0.97	+22	0.86 ± 0.076	−9

a More than 1000 cells measured. Cells were grown in LB medium at 37°C.

### Bacterial two-hybrid analysis

**Fig. 2:**
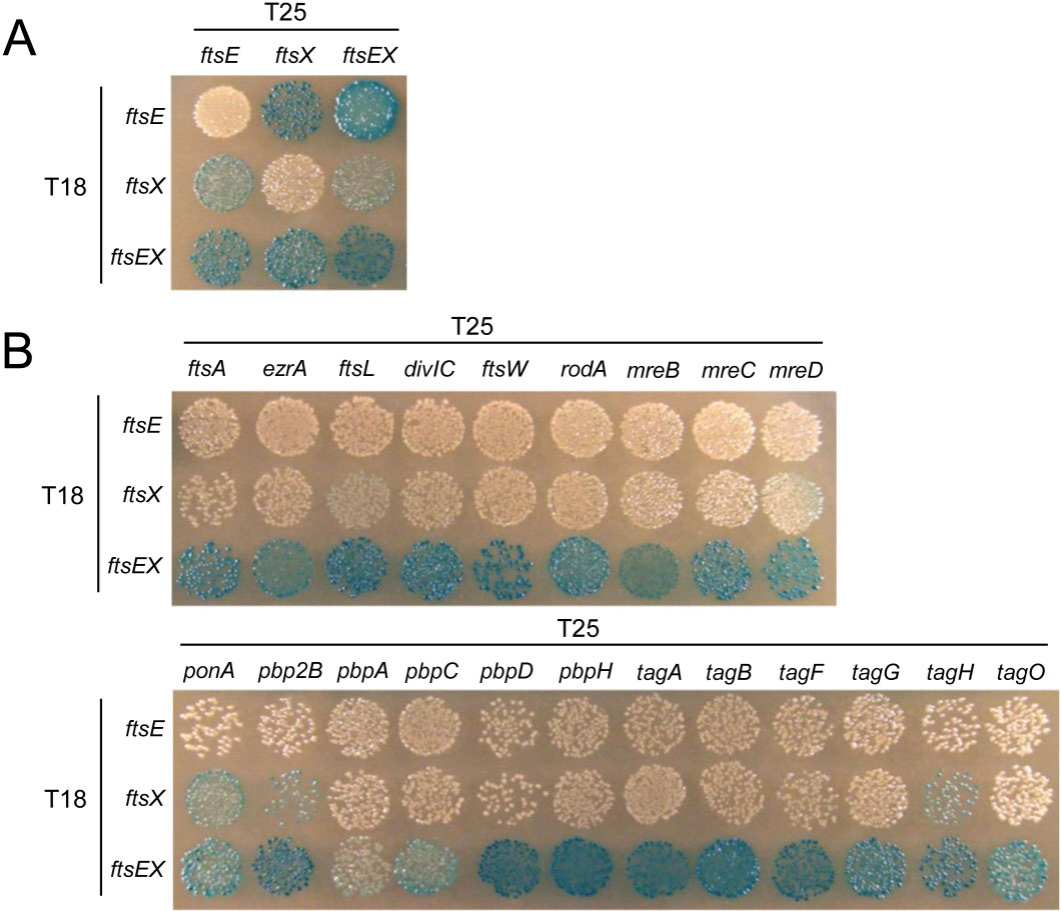
Bacterial two-hybrid analysis of FtsEX protein interactions. A. Bacterial two-hybrid analysis of interaction between FtsE and FtsX. B. Bacterial two-hybrid analysis of interaction with FtsE and FtsX. *Escherichia coli* strain BTH101 was co-transformed with two-hybrid vector plasmids (pUT18 and pKT25) expressing C-terminal fusions of the *cyaA* T18 domain to *ftsE*, *ftsX* and *ftsEX*, and N-terminal fusions of *cyaA* T25 domain to various genes, as indicated. Transformants were spotted onto nutrient agar plates containing X-Gal and incubated at 30°C for 40 h. Blue colouration indicates a positive interaction.

To explore whether the lateral wall localization and elongation phenotype was reflected in interactions of FtsE and FtsX with components of the cell wall elongation system, full-length copies of *ftsE* and *ftsX* were cloned into bacterial two-hybrid vectors (Karimova *et al*., 1998) and screened for interactions against a collection of other proteins. Figure 2A shows that FtsE and FtsX interact with each other but unlike many other cell elongation and division proteins (Wang *et al*., 1997; van den Ent *et al*., 2006; Pichoff *et al*., 2012), no self-interactions were evident. The positive interactions detected for FtsE and FtsX showed that the constructs are at least partially active.

**Fig. 3:**
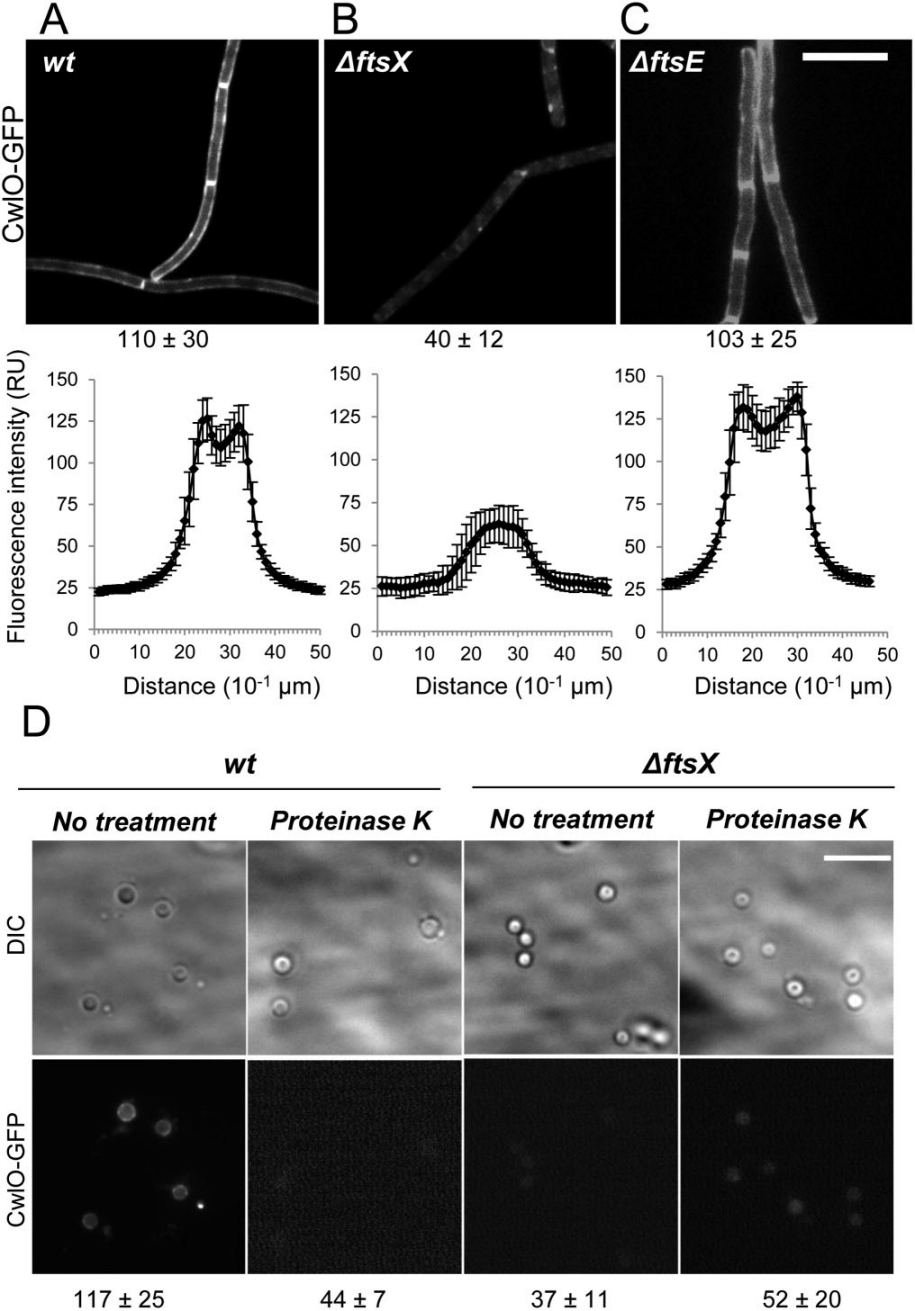
CwlO localizes at the cell membrane in an FtsX-dependent manner. A–C. Epifluorescence microscopy of strains expressing the fluorescent fusion *amyE::P_xyl_-cwlO-gfp_sf_*. The different panels correspond to (A) strain PDC528 (Bs168CA *wprA::hyg*, *epr::tet amyE::P_xyl_-cwlO-gfp_sf_*) and isogenic strains (B) PDC560 (Δ*ftsX*), (C) PDC594 (Δ*ftsE*), as indicated. Scale bar represents 5 μm. Fluorescent images were taken with the same acquisition settings and exposure times, with the maximum averaged value of quantified fluorescence intensity over the lateral wall of the cells (see Experimental procedures) indicated below. Lower panels: Profiles of fluorescence intensity corresponding to strains in (A)–(C) respectively. Averaged fluorescence intensity quantified in segments of equal size across the cell's longitudinal axis. The *y*-axis represents fluorescence intensity (relative units, RU), while the *x*-axis represents distance (10^−1^ μm). Error bars represent standard deviation of fluorescent intensity measurements. D. Cells of strains PDC528 (wt, left panels) and PDC560 (Δ*ftsX::neo*, right panels) were grown in CH media in the presence of 0.5% xylose and protoplasted (see *Experimental procedures*). Images show the relative fluorescence intensity from CwlO–GFP_sf_ protoplasts treated with proteinase K or not, as indicated. Maximum averaged value of quantified fluorescence intensities across the cells are shown below together with calculated standard deviations. Scale bar: 5 μm.

The two-hybrid constructs were then tested for interaction with various other cell wall-associated proteins (Fig. 2B). FtsE showed no strong interactions other than with FtsX. FtsX interacted significantly with three proteins tested: Pbp1a (PonA), Pbp2B and TagH. Interestingly, TagH is the ATP-binding component of the ABC transporter thought to be responsible for export of wall teichoic acid (Lazarevic and Karamata, 1995). Strikingly, when FtsE and FtsX were coexpressed on the same plasmid, interactions with many proteins involved in cell wall synthesis, cell elongation and cell division became apparent (Fig. 3B), suggesting that FtsE and FtsX need to come together before interacting efficiently with partner proteins. The interacting proteins belong to several general classes including enzymes involved in peptidoglycan (PBP) or teichoic acid (Tag) synthesis, as well as proteins implicated in elongation but with other, sometimes unknown, functions (e.g. MreCD). Several other division or cell elongation proteins, and control non-wall-associated proteins were tested, showing no evident interaction with FtsEX: FtsZ, SepF, DivIVA, PbpE, PbpX, MinC, Noc, Soj and Spo0J (not shown). The relative promiscuity of interactions for proteins involved in cell wall-associated functions has been observed previously (Mohammadi *et al*., 2007; Claessen *et al*., 2008). More work is needed to determine the extent to which these interactions occur with the native proteins in *B. subtilis* cells.

### *cwlO* and *ftsE/X* null mutants have similar cell elongation phenotypes and both are synthetic lethal with *lytE*

CwlO is the closest homologue of the FtsEX-regulated autolysins in both *E. coli* and *S. pneumoniae* (Table S1). *lytE* mutants have a different phenotype from that of *ftsEX*, having a reduced, rather than increased cell diameter (Carballido-Lopez *et al*., 2006; Fig. 1B; Table 1). The cell morphology of *cwlO* mutants has not been described in detail previously. Interestingly, under our growth conditions, *cwlO* cells had a similar phenotype to that of *ftsEX*, with short, wide and slightly undulating cells, and again this phenotype was improved by addition of 20 mM Mg^2+^ to the growth media (Fig. 1C; Table 1).

Bisicchia *et al*. (2007) previously showed that *cwlO* mutations have a synthetic lethal cell elongation phenotype when combined with *lytE*. We confirmed this result (Fig. S2A–D). To further investigate the possible connection of FtsEX with the autolytic enzymes during cell elongation we tested the effects of combining an *ftsX* deletion with null mutations in *cwlO* or *lytE*. A double-deletion mutant of *ftsX* and *cwlO* was readily constructed and did not differ in growth or morphology to either of the two single mutants (Fig. S1B). In contrast, attempts to combine *lytE* and *ftsEX* mutations were unsuccessful. We therefore generated a conditional mutant for *lytE* and introduced an *ftsX* deletion in the presence of inducer (IPTG dependent, PDC492). These cells grew in the presence but not absence of inducer (Fig. 1E). The LytE-depleted cell culture revealed that the cell chains became highly twisted and underwent extensive cell lysis (Fig. 1F and G). These phenotypic effects were similar to those of a *cwlO* deletion mutant in which *lytE* was depleted (Bisicchia *et al*., 2007), consistent with the notion that FtsEX is required for CwlO activity.

### FtsX but not FtsE is required for CwlO localization at the lateral cell wall

To test whether FtsEX determines the localization of CwlO in *B. subtilis* we expressed a CwlO–GFP_sf_ fusion in wt and *ftsE* or *ftsX* deletion strains. This took advantage of a superfolding variant of GFP (GFP_sf_) previously shown to be fluorescent after Sec-mediated transport (Dinh and Bernhardt, 2011). This protein was at least partially functional, because as the only copy of *cwlO* in cells, it was able to support growth in a *lytE* deletion strain. CwlO and LytE are both susceptible to degradation by extracellular proteases, WprA and Epr (Yamamoto *et al*., 2003; Yamaguchi *et al*., 2004; Hashimoto *et al*., 2012). Accordingly, we found that the GFP signal for CwlO was considerably enhanced by visualization in a *wprA epr* mutant background (Fig. S3B). Similarly to the results obtained by Hashimoto *et al*. (2012) (based on immunofluorescence), CwlO–GFP_sf_ was associated with the cell periphery, along the lateral cell wall, as well as at septa and cell poles (Fig. 3A). Interestingly, in a Δ*ftsX* background, the GFP fluorescence intensity was low and appeared mainly distributed throughout the cytoplasm, rather than at the cell periphery (Fig. 3B). In contrast, localization in a Δ*ftsE* mutant strain was associated with the cell periphery. It should be noted that Δ*ftsE* mutant cells were wider than those of the wild type (Fig. 3C). Fluorescence intensity measurements across typical cells (≥ 50) were plotted (Fig. 3A–C, lower panels) and these supported the peripheral localization in wild-type and Δ*ftsE* mutant cells, and the lack of CwlO recruitment to the cell envelope in the Δ*ftsX* mutant.

We then examined the localization of CwlO–GFP_sf_ after stripping the cell wall to produce protoplasts. As for intact cells, the fluorescence signal was associated with the membrane in wt cells but not in the Δ*ftsX* mutant (Fig. 3D). Treatment of protoplasts with proteinase K eliminated the surface-associated CwlO–GFP_sf_ fluorescence signal in wild-type protoplasts, whereas in the Δ*ftsX* mutant the weak cytoplasmic signal was unaffected (Fig. 3D, right panels). These experiments support the view that CwlO is exported independently of FtsEX, presumably via its classical *sec*-dependent signal peptide, and then retained on the outside surface of the cytoplasmic membrane, by a mechanism requiring FtsX.

### CwlO interacts with FtsX at the cell membrane

**Fig. 4:**
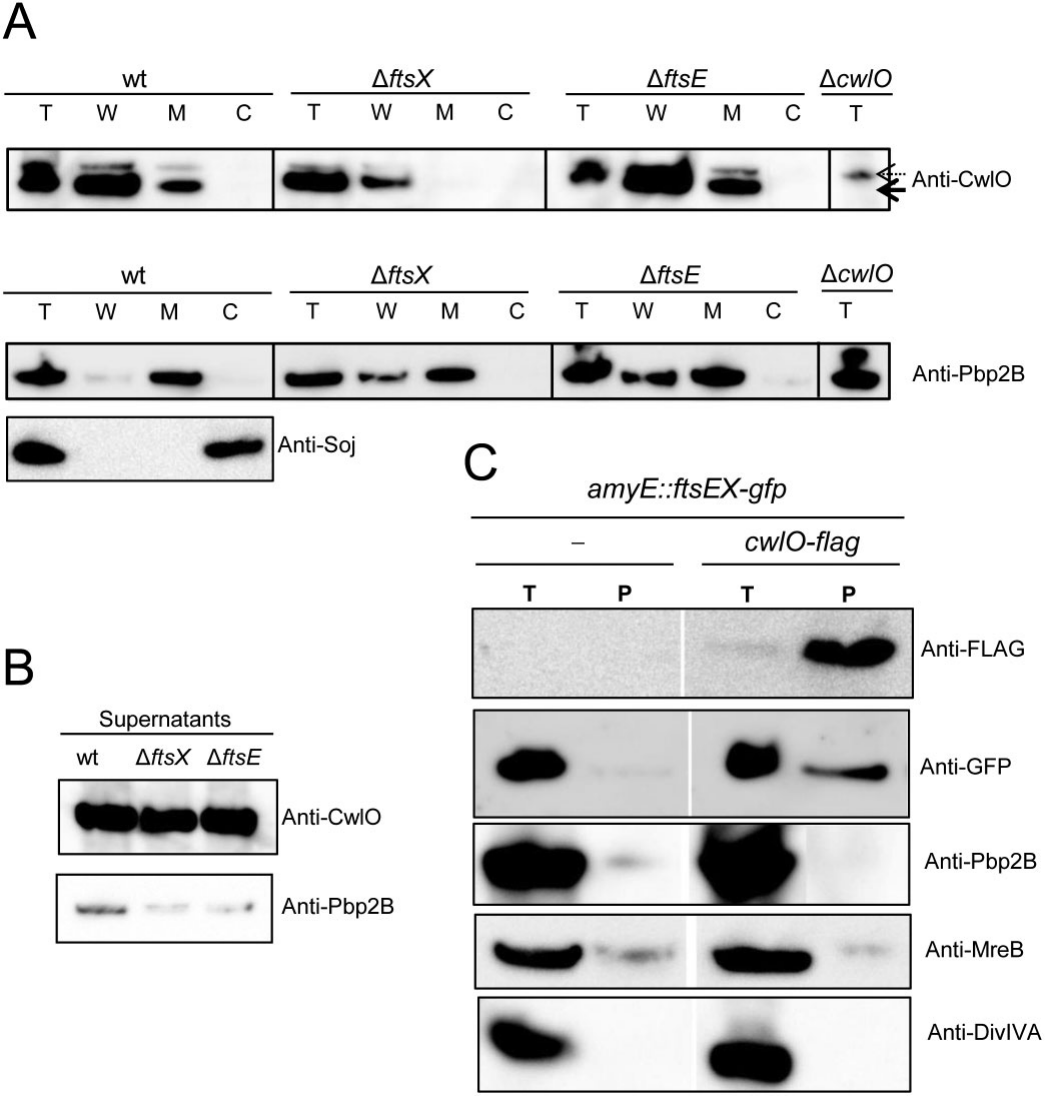
CwlO and FtsX form part of a protein complex in the cell membrane. A. Reduction in CwlO membrane fraction levels in the absence of FtsX. Fractionation of wt, Δ*ftsE* and Δ*ftsX* mutants. Total fraction from Δ*cwlO* mutant strain was analysed to discard unspecific bands. Solid black arrow indicates CwlO band after Western blotting. Dashed arrow indicates unspecific band. Lower panels correspond to cell fractionation controls using polyclonal antibodies against Pbp2B and Soj membrane and cytosolic proteins respectively. B. CwlO is detected predominantly in the supernatant fraction. C. Pull-down of CwlO-FLAG complexes in membranes of exponentially growing cells treated with formaldehyde (described in *Experimental procedures*). Bands on the Western blots were detected with anti-FLAG, anti-GFP, anti-Pbp2B, anti-MreB and anti-DivIVA antibodies, as indicated. Left lanes correspond to the control strain PDC528 were CwlO remains untagged, while expresses an FtsX–GFP fusion. Right lanes correspond to the strain PDC612 that coexpresses CwlO–FLAG and FtsX–GFP fusion proteins. T, total-cell extract prior cross-linking; P, heated pull-down fraction; the experiment was performed three times with similar results.

To investigate the role of FtsEX in CwlO function, we determined the subcellular localization of CwlO (see Supporting information) in the presence or absence of FtsEX. Cultures of wt, Δ*ftsX* and Δ*ftsE* strains were grown at 37°C. At mid-exponential phase, the cultures supernatants' (S) were collected and proteins precipitated by cold-acetone treatment; the culture's pellets were converted to protoplasts by incubation with lysozyme. The protoplasts were collected by centrifugation, leaving a supernatant that constituted the cell wall (W) fraction. Cell membranes (M) and cytoplasmic fractions (C) were obtained from the protoplast pellets (see *Experimental procedures*). The untreated half of the culture pellet constituted the total (T) fraction. In wt and Δ*ftsE* cells, CwlO, was detected in both the membrane and cell wall fractions (Fig. 4A). However, in Δ*ftsX* mutant cells, the CwlO signal was absent from the membrane fraction and reduced in the wall fraction (Fig. 4A). As controls, we examined the distribution of a well-characterized cytosolic protein Soj (Fig. 4A), and an integral membrane protein Pbp2B (Fig. 4A), which were indeed detected predominantly in the cytoplasmic or membrane fractions respectively. When we analysed the presence of CwlO in the supernatant fraction (Fig. 4B), the protein was detected in all three backgrounds in large amounts.

To further investigate the interaction between FtsEX and CwlO we used chemical cross-linking followed by pull-down of FLAG-tagged CwlO, then tested for the presence of FtsX protein (Fig. 4C). As expected CwlO-FLAG was only detected in samples containing the *cwlO-flag* construct. In these samples a single band corresponding to the expected molecular weight (53 KDa) was detected in cross-linked samples after heating (Fig. 4C). In non-heated samples a very faint band was sometimes detected with similar mobility, but no other bands corresponding to high-molecular-weight complexes were detected in the Western blots. Silver staining did reveal a prominent complex with a mass > 100 KDa (Fig. S4). Importantly, anti-GFP anti-serum detected the FtsX–GFP fusion protein in the pull-down samples, but only in the presence of CwlO-FLAG (Fig. 4C). Three other tested cytosolic or membrane-associated proteins, Pbp2B, MreB and DivIVA, were not detected in the pull-down samples. These results suggest that CwlO associates, directly or indirectly, with the membrane component of the ABC transporter, FtsX.

### Differential roles of MreB isoforms in control of autolytic activity

We previously reported that LytE interacts with MreBH and that its localization in the lateral cell wall is at least partly dependent on this interaction (Carballido-Lopez *et al*., 2006). We wondered whether one or other of the MreB isologues control CwlO localization or activity. We recently showed that strains containing only one of the three homologues can be obtained, provided that the remaining protein is overproduced. Availability of these strains provided a means of testing whether the roles of the MreB homologues are differentiated in respect of the control of CwlO or LytE activities. We therefore introduced conditional mutations of the *ftsEX* or *lytE* genes into strains expressing single *mreB* isologues.

**Fig. 5:**
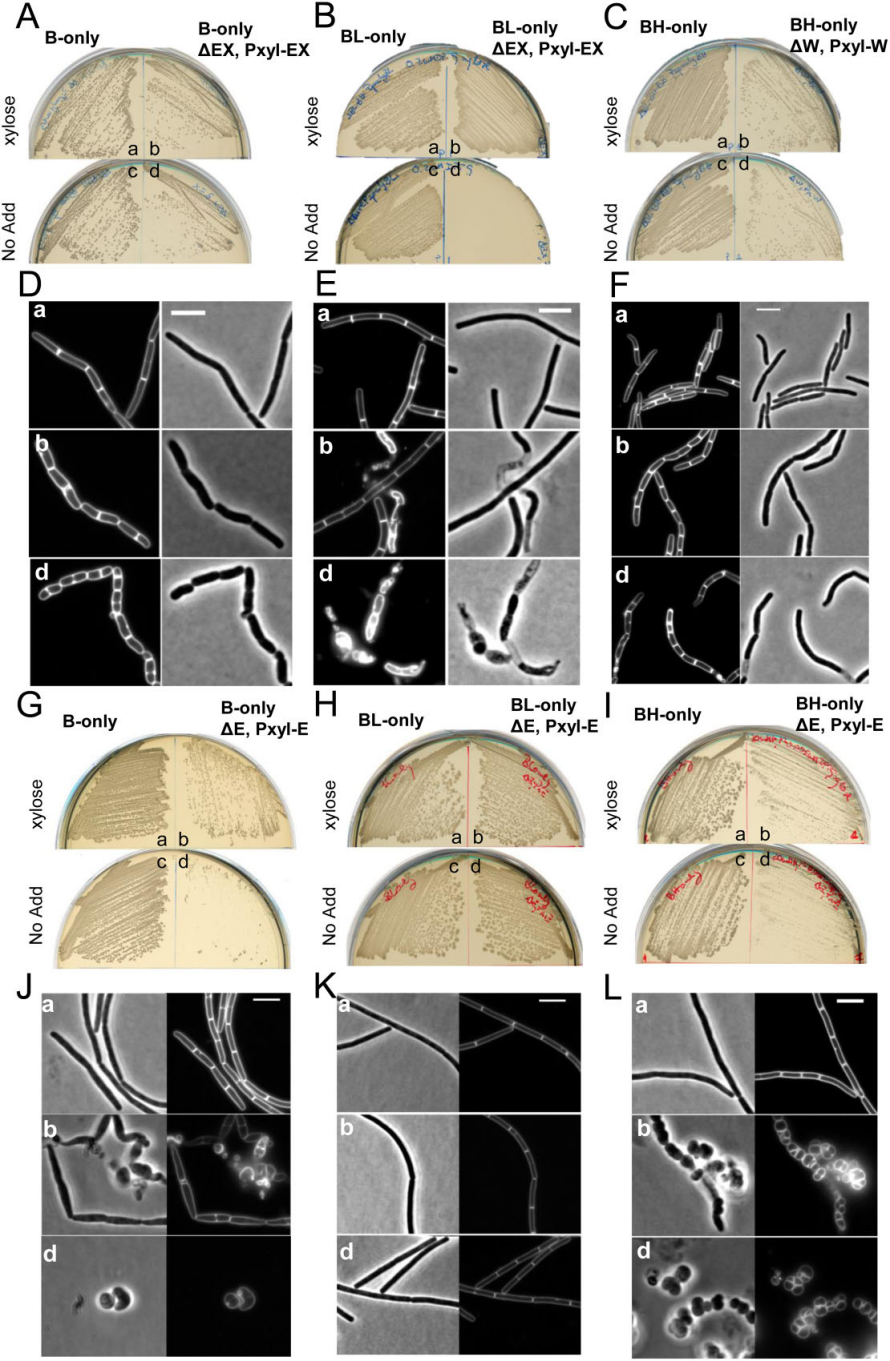
CwlO activity depends on the Mbl actin homologue. A–C. Growth of strains B-only, BL-only and BH-only (a/c), respectively, and its derivatives PDC664, PDC643 and PDC659 (b/d) on NA 20 mM Mg^2+^, supplemented with appropriate concentration of IPTG in each case (Kawai *et al*., 2009a), in the presence (a–b) or absence of xylose (c–d), as indicated. D–F. Cell morphologies of typical fields of strains in (A)–(C) respectively. Phase-contrast and membrane-stained fluorescent images (FM5-95) of parental strains (a) and its derivatives in the presence (b) or absence (d) of xylose for 3 h at 37°C. G–I. Growth of strains B-only, BL-only and BH-only (a/c), respectively, and its derivatives (Δ*lytE::spec*, *aprE::P_xyl_-lytE*) PDC688, PDC678 and PDC697 (b/d) on NA 20 mM Mg^2+^, supplemented with appropriate concentration of IPTG in each case (Kawai *et al*., 2009a), in the presence (a–b) or absence of xylose (c–d). J–L. Cell morphologies of typical fields of strains in (G)–(I) respectively. Phase-contrast and membrane-stained fluorescent images (FM5-95) of parental strains (a) and its derivatives in the presence (b) or absence (d) of xylose for 3 h at 37°C.

The most striking results were obtained for the Mbl-only strain. When a conditional allele of *lytE* was introduced, the Mbl-only strain showed virtually normal growth, independent of *lytE* expression (Fig. 5H and K). This result shows that a strain with just Mbl can presumably support the activity of the FtsEX/CwlO system, because this is essential when LytE is not present. In contrast, when *ftsEX* was depleted in this strain (Fig. 5B and E) growth was abolished and extensive cell lysis occurred. This suggests that Mbl is not able to support efficiently the activity of LytE during cell elongation, and is therefore specific for FtsEX/CwlO.

Strikingly, the results for the MreB- and MreBH-only strains were, in both cases, reciprocal to those obtained for the Mbl-only strain: thus, cell growth and morphology was much more affected in the absence of *lytE* (Fig. 5G, I, J and L) than *ftsEX* (Fig. 5A, C, D and F).

We also examined the effects of combining *lytE, cwlO* or *ftsEX* mutations with single deletions of each *mreB* homologue. In general, the results were consistent with those described above (Fig. S5), although they were less clear cut, as expected, because each of these strains still contains two *mreB* isologues. Nevertheless, it was clear that the effects of an *mbl* mutation were greatly exacerbated by *lytE* mutation but not significantly by *cwlO* or *ftsEX* mutation, whereas *mreB* or *mreBH* mutants tended to show the opposite effect. All of the data support the idea that Mbl is crucial for functioning of the FtsEX/CwlO system, whereas MreB and MreBH are more important for LytE action.

## Discussion

### FtsEX regulates the CwlO autolysin required for cell elongation in *B. subtilis*

Previous work on the FtsEX systems of *E. coli* and *S. pneumoniae* have demonstrated an unexpected role for the FtsEX ABC-transporter-like protein complex in regulation of specific cell wall autolytic enzymes (Sham *et al*., 2011; Yang *et al*., 2011). Our work establishes that FtsEX of *B. subtilis* has an analogous role, albeit that this system seems to be involved mainly or exclusively with the cell elongation system, rather than cell division. Various phenotypic and functional properties of the FtsEX system, reported here and previously (Garti-Levi *et al*., 2008) are consistent with a role in elongation: the mutants are shorter and wider than wild-type cells; the phenotype is rescued by high Mg^2+^ concentrations; the proteins localize in the lateral wall; and the cognate autolysin, CwlO, also seems to be involved in cell elongation.

**Fig. 6:**
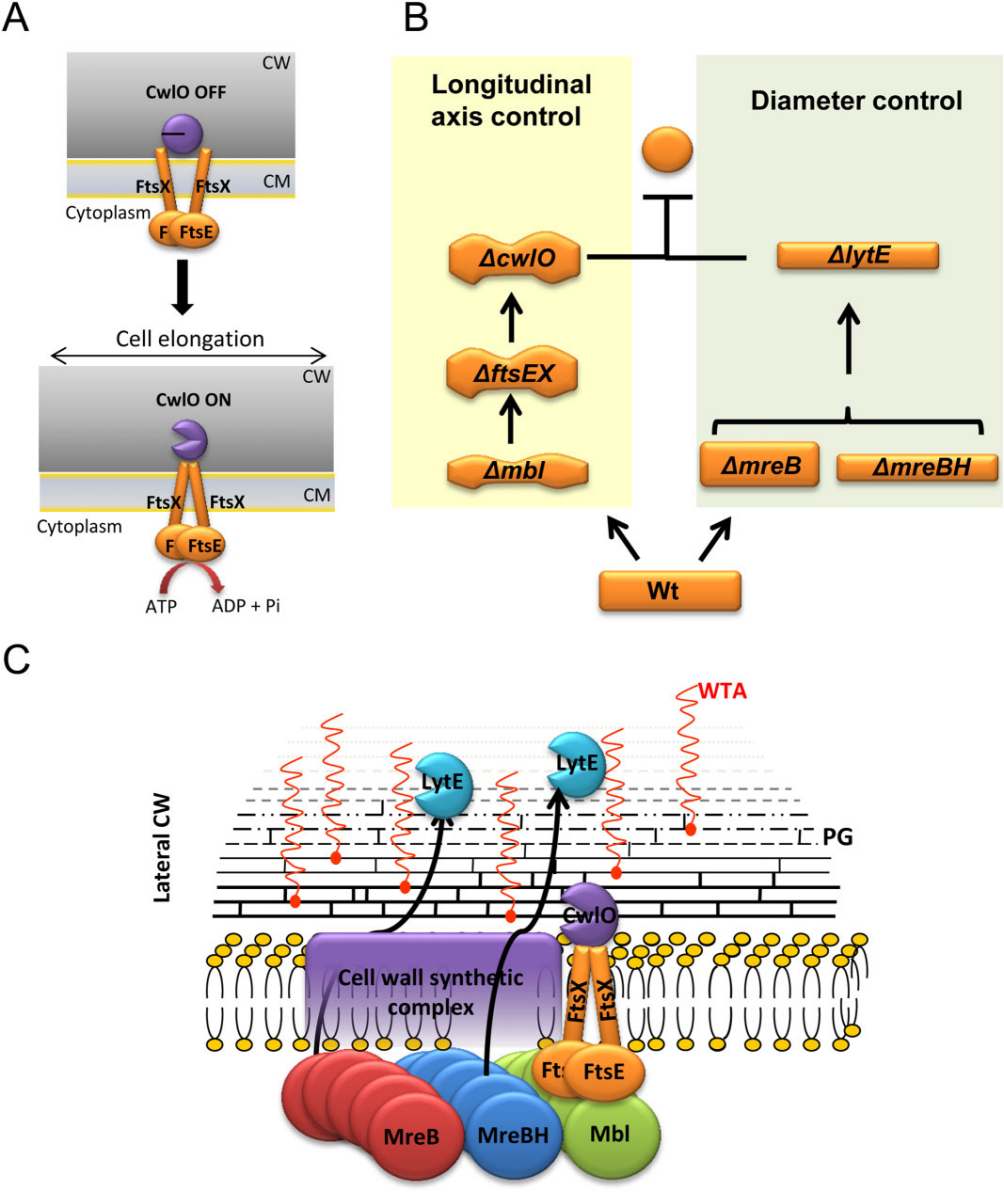
Model for actin cytoskeleton function in co-ordination of CW hydrolytic activities CwlO and LytE during cell elongation. A. Schematic representation of the FtsEX ATPase cycle during activation of the CwlO CW hydrolase activity in *B. subtilis*. FtsEX is shown in the model as a tetramer, although other stoichiometries are possible on the basis of the two-hybrid data. B. Two distinct pathways for CW hydrolytic activity at the lateral cell wall in *B. subtilis*. C. Co-ordination by the actin cytoskeleton to ensure the balance between cell wall synthesis and hydrolysis during cell elongation. See text for further details.

The similarity of phenotypes of *ftsEX* and *cwlO* mutants is also consistent with FtsEX regulating CwlO activity, as is the synthetic lethality of *ftsEX* with *lytE*, just as for *cwlO* and *lytE* (Bisicchia *et al*., 2007; Hashimoto *et al*., 2012). Biochemical experiments also support the notion that FtsEX regulates CwlO: first, we found that CwlO is sequestered to the external surface of the cytoplasmic membrane by a mechanism requiring FtsX; second, protein interactions were detected by both cross-linking and pull-down and cell fractionation experiments. The results lead to a model in which FtsEX regulates CwlO activity by a direct protein–protein interaction (Fig. 6A), similar to those described previously for other organisms (Sham *et al*., 2011; Yang *et al*., 2011).

### Specialized functional roles for MreB isologues in regulation of cell wall autolytic activities

Bacterial actin homologues of the MreB family are thought to orchestrate the synthesis and assembly of the cell wall in most rod-shaped bacteria. The proteins are present in all major lineages of bacteria, as are the genes for cell wall synthesis, suggesting that both the wall and its regulation by MreB were present in bacteria very early in evolution (Trachtenberg, 1998; Carballido-Lopez and Errington, 2003b; Margolin, 2009). Many bacteria have multiple MreB isologues and *B. subtilis* has three (Jones *et al*., 2001; Carballido-Lopez *et al*., 2006). The isologues are thought to have overlapping but differentiated functions, but molecular details of how these functional specializations are achieved remain poorly understood.

Initial attempts to examine the possible involvement of MreB proteins in the FtsEX system were inconclusive because single mutants for any one isologue have two remaining isologues with overlapping and/or complementary functions. However, by taking advantage of recently developed strains containing single MreB isologues (Kawai *et al*., 2009a) more incisive results were obtained. In particular, it was evident that a strain containing only Mbl (but not MreB- or MreBH-only) grew normally in the presence of the FtsEX/CwlO system. Depletion of its expression resulted in massive cell lysis. In contrast, depletion of *lytE* showed no phenotypic effect in the Mbl-only strain. This strongly suggests that Mbl can support functioning of the FtsEX/CwlO system but that it cannot support LytE function. The MreB- and MreBH-only strains did not have such clear-cut phenotypes but, in both cases, the results were complementary to the ones obtained with the Mbl-only strain. They tolerated loss of FtsEX or CwlO well, but were extremely sick in the absence of LytE. These results suggest that the MreB homologues are highly differentiated in respect of the control they exert over the FtsEX/CwlO and LytE autolytic systems, with Mbl specialized for regulation of the former and MreB and MreBH for the latter (Fig. 6B).

These results additionally show that the MreB proteins are pivotally involved in the regulation of autolytic activity, in addition to their previously demonstrated roles in control of peptidoglycan and wall teichoic acid synthesis (Formstone *et al*., 2008; Yamamoto *et al*., 2008; Kawai *et al*., 2009b; 2011). They also provide the clearest example so far of how control of different specific enzymes is the likely explanation for the presence of multiple MreB isologues in many bacteria.

### Differentiated roles for the CwlO and LytE autolysins in different aspects of cell morphogenesis

It is now well recognized that most bacteria have multiple autolytic enzymes and the presumption is that they are involved in different aspects of cell wall synthesis or remodelling. However, in only a few cases are the specific roles for the autolysins understood. Recent work in *B. subtilis* and *E. coli* has shown that the autolysins involved in cell elongation of both organisms are partially redundant, with two proteins of *B. subtilis*, LytE and CwlO (Hashimoto *et al*., 2012), and three of *E. coli*, Spr, YdhO and YebA (Singh *et al*., 2012) having overlapping functions. Lethal phenotypes are revealed only when both or all of the redundant autolysin genes are deleted (Bisicchia *et al*., 2007; Singh *et al*., 2012). Our results provide important new insights into the specialized roles of LytE and CwlO. LytE function is complicated by the fact that it is involved not only in elongation but also in cell separation (Yamamoto *et al*., 2003; Fukushima *et al*., 2006). We previously reported that *lytE* mutants, in addition to forming slightly longer chains of cells, are also slightly thinner than wild-type cells. The basis for this thinning is not clear, although it is interesting that this phenotype is shared by other cell elongation mutants, including *ponA* (encoding a major peptidoglycan synthase; Murray *et al*., 1998; Kawai *et al*., 2009b) and *mreBH* (which has some role in regulation of LytE localization; Carballido-Lopez *et al*., 2006). In this article we report that *cwlO* mutants are different (but similar to *ftsEX* mutants) in being wider than the wild type as well as being slightly bent or undulated (Fig. 6B). These distinct phenotypes suggest that the proteins have differentiated roles in cell morphogenesis during growth. Irrespective of how they work, one or other activity must be present or, as shown previously and herein, cells stop elongating altogether (Bisicchia *et al*., 2007; Fig. 1D–G).

The model shown in Fig. 6C illustrates one important difference between the likely roles of CwlO and LytE. Because CwlO is activated by the membrane protein complex FtsEX, its activity is probably restricted to the inner part of the thick (∼ 50 nm) Gram-positive cell wall. Furthermore, requirement for Mbl function suggests that CwlO activity might also be tightly co-ordinated with that of the various peptidoglycan and teichoic acid synthases. Thus, CwlO might contribute intimately to the insertion of newly synthesized wall material, along with the many other proteins associated with the MreB system. In contrast, LytE is probably regulated by some other mechanism. Traditionally, it has been assumed that in Gram-positive bacteria, the multi-layered wall material matures as it migrates outwards, progressively underlain by newly inserted material. The outer layer(s) is thought to be stretched and load bearing, requiring autolytic activity to enable cell growth (Holtje, 1996b; Holtje, 1998; Hayhurst *et al*., 2008). Although LytE insertion into the wall is probably again co-ordinated with that of wall synthesis by interaction with MreBH and possibly MreB, it has a wall-binding domain that could facilitate its migration outwards during wall maturation. Also, LytE synthesis is regulated in response to various stresses (Bisicchia *et al*., 2007; Schirner and Errington, 2009; Salzberg *et al*., 2013). Thus, we envisage the LytE function more as a stress response factor that is synthesized and or recruited when wall expansion is compromised.

We suggest that when CwlO is the major elongation autolysin (*lytE* mutant), PG synthesis is well co-ordinated with turnover and the normal pattern of growth is achieved, albeit that the cells are narrower than normal. In contrast, when LytE is the only major autolysin, regulated growth and turnover is impaired but LytE activity enables growth albeit in a relatively disordered manner, leading to loss of control over cell width and ability to maintain a consistent longitudinal axis of growth. The differentiation of these two cell phenotypes is reminiscent of the different phenotypes generated by mutations affecting the major MreB isologues, MreB and Mbl (Fig. 6B). As reported some years ago, *mbl* mutants have a distinctive highly twisted phenotype, whereas *mreB* mutants tend to maintain control over longitudinal growth, albeit becoming much wider than the wild type (Abhayawardhane and Stewart, 1995; Jones *et al*., 2001). It now appears that differences in the control of autolytic activity might contribute significantly to these distinct phenotypes. *mreB* mutants retain Mbl function and therefore FtsEX/CwlO activity, and thereby are still able to regulate PG synthesis and turnover in a highly co-ordinated way, whereas *mbl* mutants rely on the LytE system which is less tightly co-ordinated with the synthetic machinery. Our results therefore shed new light on the differentiated roles of MreB isologues in cell morphogenesis and about the specialized roles of two major autolytic proteins in the regulation of cell shape.

## Experimental procedures

Complete details of all the experimental procedures used are provided in Supporting information.

### Bacterial strains and plasmids, and primers

The bacterial strains, plasmids and oligonucleotides sequences used in this study are listed in Tables S2–S4 respectively. All *B. subtilis* strains used in the reported experiments are derivatives of Bs168CA. The construction of plasmids is described in Supporting information.

### Growth conditions and media

Nutrient agar (NA, Oxoid) was used for routine selection and maintenance of both *B. subtilis* and *E. coli* strains. For *B. subtilis*, cells were grown in Luria–Bertani (LB), CH or SMM defined minimal medium (Anagnostopoulos & Spizizen) containing 0.5% xylose or 1 mM IPTG when required, unless stated otherwise. For *E. coli*, cells were grown in LB medium. Supplements and antibiotics were added as required: 20 μg ml^−1^ tryptophan, 100 μg ml^−1^ ampicillin, 5 μg ml^−1^ chloramphenicol, 5 μg ml^−1^ kanamycin, 50 μg ml^−1^ spectinomycin, 0.75 μg ml^−1^ erythromycin and 10 μg ml^−1^ tetracycline.

### Microscopic imaging

For fluorescence microscopy, cells were grown to mid-exponential phase at 30°C or 37°C and mounted on microscope slides covered with a thin film of 1.2% agarose. See figure legends for specific growth conditions employed for each experiment. Fluorescence microscopy was carried out using Zeiss Axiovert 200M, Nikon Eclipse Ti-U, spinning disk confocal microscope. The images were acquired with Metamorph 6 (Molecular Devices) and FRAP-AI 7 (MAG Biosystems) software, and analysed using ImageJ v.1.44o (National Institutes of Health). When required, cells were incubated in the presence of the membrane dye FM5-95 (90 μg ml^−1^, Molecular Probes) prior to microscopic examination.

### Sample preparation for microscopy

For sample preparation, overnight pre-cultures of *B. subtilis* were grown in CH medium supplemented with 20 mM MgSO_4_ (CH-Mg) and appropriate antibiotic selection, from freshly isolated colonies on plates. Day cultures were performed by diluting pre-culture to an OD_600_ of 0.02 in CH-Mg and grown at 30°C. Expression of fluorescent CwlO–GFP_sf_ fusion was induced by addition of 0.3% xylose. Samples for microscopic observation were taken at mid-exponential phase and immobilized on 1.2% agarose-coated microscope slides.

### Protoplast preparation for microscopy

Cells of strains PDC528 (wt, CwlO–GFP_sf_) and PDC560 (Δ*ftsX::neo*, CwlO–GFP_sf_) were grown in CH media in the presence of 0.5% xylose. Cells were harvested and resuspended in CH-MSM media in the presence of 0.5% xylose. Cells were protoplasted by incubation with 0.5 mg ml^−1^ lysozyme during 30 min at 30°C. After CW digestion, the protoplasts suspensions were split in two. One half was treated with proteinase K (10 μg ml^−1^) for 30 min.

### Two-hybrid analysis

To screen for interactions of FtsX and FtsE with various proteins involved in cell wall synthesis or cell division, the *ftsX*, *ftsE* and *ftsEX* coding sequences were amplified by PCR and cloned into two bacterial two-hybrid vectors (pUT18 and pKT25), resulting in a C-terminal fusion of the *cyaA* (adenylate cyclase) T18 domain, or an N-terminal fusion of the *cyaA* T25 domain respectively (Karimova *et al*., 1998). In addition, the coding sequences of *ftsA, ezrA, ftsL, divIC, ftsW, rodA, mreB, mreC, mreD, ponA, pbp2B*, *pbpA, pbpC, pbpD, pbpH, tagA, tagB, tagF, tagG, tagH, tagO* were amplified by PCR and cloned into pKT25 generating N-terminal fusions of the T25 domain. Finally, to test the putative interaction between the FtsX extracytoplasmic loop1 and the CW hydrolase CwlO, plasmid pairs encoding the FtsX extracytoplasmic loop1 and either the full-length CwlO or the N-terminal coil-coiled domain coding sequences were co-transformed into BTH101 (cya-99). Co-transformants were spotted onto nutrient agar or minimal media plates, as indicated, containing ampicillin (100 μg ml^−1^), kanamycin (25 μg ml^−1^) and 0.004% X-gal. Pictures were taken after 40–72 h of growth at 30°C. Under these conditions, control transformations with empty vectors remained white for up to 72 h of incubation.

### Cell fractionation and immunoblotting

*Bacillus subtilis* cell cultures were grown in LB at 37°C. When cells reached mid-exponential phase, cultures (100 ml) were collected by centrifugation (8000 *g* for 10 min at 25°C). Culture supernatants' protein content (S) was recovered by cold-acetone precipitation. Pellets were resuspended in 4 ml 1× SMM buffer (0.5 M sucrose, 20 mM MgCl_2_, 20 mM maleic acid, pH 7); 250 μl 10 mg lysozyme ml^−1^ (Sigma) and 50 μl complete protease inhibitor (EDTA-free, Roche) were added to cell suspensions and incubated at 37°C for 1 h with gentle shaking. Then cultures were split into two (2 × 2 ml). First half constituted the total fraction (T). The second half was used to obtain the cell wall (CW), membrane (M) and cytoplasmic (C) fractions, as described in detail within Supporting information. Ten micrograms of total protein from each extract were separated by SDS-PAGE on a 4–12% gradient gel (Novex, Life technologies). Separated proteins were analysed by Western blotting as described in detail in Supporting information.

### Formaldehyde cross-linking and pull-down of CwlO complexes

Cross-linking and pull-down experiments were performed with some modifications as described by Sham *et al*. (2011). Briefly, cultures (400 ml) of strains PDC612 (Bs168CA Δ*wprA::hyg* Δ*epr::tet ΩcwlO-FLAG amyE::P_xyl_-ftsEX-gfp*) and PDC613 (Bs168CA Δ*wprA::hyg* Δ*epr::tet amyE::P_xyl_-ftsEX-gfp* parent negative control) were grown exponentially to OD_600_ of 0.5. Cells were collected by centrifugation (8000 *g* for 10 min at 25°C). Cell pellets were washed with 18 ml 1× PBS at 25°C, and cells were collected again by centrifugation. Residual supernatants were removed. Washed pellets were suspended in 19 ml 1× PBS, to which 1200 μl 37% of formaldehyde solution (Sigma) were added. Mixtures were incubated at 37°C for 1 h. Cross-linking reactions were quenched by the addition of 4 ml 1.0 M glycine followed by incubation for 10 min at 25°C. Cells were collected by centrifugation, washed with 20 ml 1× PBS at 25°C and centrifuged again. Pull-down of CwlO-FLAG complexes was performed using an anti-FLAG M2 affinity gel, as described previously (Sham *et al*., 2011). Identification of protein content in the different fractions was carried out by Western blotting with appropriate anti-sera. A full protocol description can be found within Supporting information.
