# Supplementary material for: Differentiated roles for MreB-actin isologues and autolytic enzymes in Bacillus subtilis morphogenesis
Source: Mol Microbiol. 2013 Aug 4;89(6):1084–98. doi: 10.1111/mmi.12335 (PMC3817527; doi:10.1111/mmi.12335)
Supplement: Supplementary file 1 [file mmi0089-1084-SD1.pdf]

## Supporting information for:

# Differentiated roles for MreB-actin isologues and autolytic enzymes in *Bacillus subtilis* morphogenesis

Patricia Domínguez-Cuevas*, Ida Porcelli[1], Richard Daniel and Jeff Errington*

Centre for Bacterial Cell Biology. Newcastle University. Newcastle upon Tyne. UK

[1] Present address: Institute of Food Research, Norwich Research Park, Colney lane, Norwich, NR4 7UA

* Corresponding authors. Patricia Domínguez-Cuevas. E-mail: Patricia.Dominguez-Cuevas@ncl.ac.uk or Jeff Errington. E-mail: jeff.errington@ncl.ac.uk. Centre for Bacterial Cell Biology. Newcastle University. Baddiley-Clark Building. Richardson Road. NE2 4AX. Newcastle upon Tyne. UK. Tel.: +44 (0) 191 208 3232; Fax: +44 (0) 191 208 3237

**Supplementary Experimental procedures**
**Supplementary Figures:**
**Figure S1.** Phenotypes associated with *ftsX* and *cwlO* mutant strains.
**Figure S2.** LytE and CwlO mutants are synthetically lethal.
**Figure S3.** CwlO localization in different genetic backgrounds.
**Figure S4.** FtsX and CwlO interact within the same protein complex at the cell membrane.
**Figure S5.** LytE is synthetically lethal with Mbl
**Supplementary Tables:**
**Table S1**. E-values and identity homologies for FtsEX and the CW hydrolases CwlO and LytE
**Table S2.** Bacterial strains
**Table S3**. Plasmids
**Table S4**. Primers
**Supplementary References**

**Supplementary Experimental procedures**

***General methods***

DNA manipulations and *E. coli* DH5α transformations were carried out using standard methods (Sambrook *et al.*, 1989). Plasmid DNA and PCR fragments were purified using the Qiaprep spin miniprep kit (Qiagen) or the Qiaquick PCR purification kit (Qiagen), respectively. Transformation of competent *B. subtilis* cells was performed using an optimized two-step starvation procedure as previously described (Anagnostopoulos & Spizizen, 1961; Hamoen *et al.*, 2002). Nutrient agar (NA, Oxoid) was used for routine selection and maintenance of both *B. subtilis* and *E. coli* strains. For *B. subtilis*, cells were grown in Luria–Bertani (LB), CH or SMM defined minimal medium (Anagnostopoulos & Spizizen) containing 0.5% xylose or 1 mM IPTG when required, unless stated otherwise. For *E. coli*, cells were grown in LB medium. Supplements were added when necessary as required: 20 µg $ml^{-1}$ tryptophan, 100 µg $ml^{-1}$ ampicillin, 5 µg $ml^{-1}$ chloramphenicol, 5 µg $ml^{-1}$ kanamycin, 50 µg $ml^{-1}$ spectinomycin, 0.75 µg $ml^{-1}$ erythromycin and 10 µg $ml^{-1}$ tetracycline.

***Strain construction***

***<u>Deletion strains</u>***

Deletion of the *ftsE, ftsX* and *ftsEX* (*B. subtilis* strains 4503, 4501 and 4502, respectively) was accomplished by double crossover of a kanamycin marker. Regions of DNA upstream and downstream of *ftsE, ftsX or ftsEX* (~3 kb each side) were PCR-amplified, using primers RevA1/RevA2 and ForA, and ForB1/ForB2 and RevB, respectively (Table S4). The kanamycin/neomycin marker was cut out of plasmid pBEST501. The PCR fragments and the kanamycin/neomycin marker were digested, ligated, and the ligation product was directly transformed to competent Bs168CA cells. All chromosomal integrations were verified by PCR, restriction digestion, and sequencing.

***<u>Fluorescent fusions</u>***

**CwlO-GFP$_{sf}$**

*GFP$_{sf}$* was amplified by PCR from the pUC57-GFP$_{sf}$ plasmid DNA using primers GFP-sf-FEcoRI and GFPsf-RevSpeI-NotI, and then cloned between the EcoRI and NotI sites of plasmid pSG1728-CwlO, creating pSG-cwlO-gfp$_{sf}$. The resulting plasmid was used to transform Bs168CA *ΔwprA Δepr* (PDC538), with selection for spectinomycin resistance, to generate the strain PDC528, in which the *gfp$_{sf}$*-fused to *cwlO* is expressed from the xylose inducible promoter $P_{xyl}$ at the *amyE* locus on the *B*. s*ubtilis* chromosome. Disruption of *amyE* was confirmed using a starch plate assay (Cutting, 1990), and the correct integration of the inserts at the *amyE* locus was confirmed by PCR. The GFP variant used was superfolder GFP (Pedelacq *et al.*, 2006), which is being shown to be functional in the periplasm following Sec export (Dinh & Bernhardt, 2011).

**FtsEX-GFP$_{sf}$**

*GFPsf* was amplified by PCR from the pUC57-GFPsf plasmid DNA using primers GFP-sf-FEcoRI and GFPsf-RevSpeI-NotI, and then cloned between the EcoRI and NotI sites of plasmid pSG1728-ftsEX, creating pSG-ftsEX-gfp$_{sf}$. The resulting plasmid was used to

transform Bs168CA, with selection for spectinomycin resistance, to generate the strain PDC534, in which the $gfp_{sf}$-fused to *ftsX* is expressed from the xylose inducible promoter $P_{xyl}$ at the *amyE* locus on the *B*. s*ubtilis* chromosome. Disruption of *amyE* was confirmed using a starch plate assay, and the correct integration of the inserts at the *amyE* locus was confirmed by PCR.

***Epitope tagging***

**CwlO-Flag**

To construct pMUTin-'cwlO-flag a fragment containing the last 550 bp of the *cwlO* orf was amplified by PCR with the primers cwlOCterFHindIIIFlag and cwlOrevKpnIFlag from the wild-type strain 168 genomic DNA, digested with HindIII and KpnI, and inserted into the corresponding HindIII-KpnI sites of pMUTin-flag plasmid. The resulting plasmid was used to transform PDC538, with selection for erythromycin resistance, to generate the strain PDC609, in which the flag-fused to *cwlO* is expressed from the native promoter at the *native cwlO* locus on the *B*. s*ubtilis* chromosome. The newly generated strain showed a wt phenotype, indicating that the flag fusion is fully functional. The correct integration of the plasmid at the native locus was confirmed by PCR.

***Inducible expression strains***

***amyE::$P_{xyl}$-cwlO***

*cwlO* was amplified by PCR from the wild-type strain 168 genomic DNA using primers CwlO-FXhoI and cwlORev-EcoRI, then cloned between the *Xho*I and *EcoRI* sites of plasmid pSG1728, creating pSG1728-cwlO. The resulting plasmid was used to transform Bs168CA, with selection for spectinomycin resistance, to generate the strain PDC567, in which *cwlO* is expressed from the xylose inducible promoter $P_{xyl}$ at the *amyE* locus on the *B*. s*ubtilis* chromosome.

***aprE::$P_{spac}$-lytE***

*lytE* was amplified by PCR from the wild-type strain 168 genomic DNA using primers LytEFXmaI and LytERevEcoRI, then cloned between the *XmaI* and *EcoRI* sites of plasmid pAPNC213-erm, creating pAPNerm-$P_{spac}$-lytE. The resulting plasmid was used to transform Bs168CA, with selection for erythromycin resistance, to generate the strain PDC620, in which *lytE* is expressed from the IPTG inducible promoter $P_{spac}$ at the *aprE* locus on the *B*. s*ubtilis* chromosome. The correct integration of the inserts at the *aprE* locus was confirmed by PCR.

***aprE::$P_{xyl}$-cwlO, aprE::$P_{xyl}$-ftsEX and aprE::$P_{xyl}$-lytE***

*cwlO*, *ftsEX* and *lytE* orfs were amplified by PCR from the corresponding plasmids pSG-$P_{xyl}$-cwlO, pSG-$P_{xyl}$-ftsEX-$gfp_{sf}$ and pSG-$P_{xyl}$-$P_{wt}$-LytEmcherry, respectively, using primers $P_{xyl}$-FsphI and amyEtoAprERevBamHI (or LytE-revSacI, in the case of LytE), then cloned between the SphI and BamHI (or SacI, in the case of LytE) sites of plasmid pAPNC213-ery, creating the plasmids pAPNC-$P_{xyl}$-cwlO, pAPNC-$P_{xyl}$-ftsEX and pAPNC-$P_{xyl}$-Pwt-LytE. The resulting plasmids were used to transform the corresponding deletion mutant strains, with

selection for erythromycin resistance, to generate strains PDC639, PDC635 and PDC702 respectively, in which the different *orfs* are expressed from the xylose inducible promoter $P_{xyl}$ at the *aprE* locus on the *B*. *subtilis* chromosome. The correct integration of the inserts at the *aprE* locus was confirmed by PCR.

**B-only, BL-only and BH-only strains (PDC660, YK1119 and PDC643)**

BL-only strain (YK1119) was already constructed as described in Kawai *et al.,* 2011. *B. subtilis* strain YK1012 (*Δmbl ΔmreBH*) was transformed with chromosomal DNAs corresponding to *amyE* insertions of overexpression constructs for *mreB* and *mreBH*, with selection for chloramphenicol resistance (*amyE::*$P_{spacHY}$*-mreB* and *amyE::*$P_{spacHY}$*-mreBH*), respectively (Kawai *et al.*, 2011). The resulting strains were subsequently transformed with chromosomal DNA from strain YK1119, with selection for kanamycin resistance on NA plates supplemented with 20 mM $Mg^{+2}$.

Resulting strains PDC660, YK1119 and PDC643 were then transformed with chromosomal DNA from strains PDC639, PDC635 and PDC702 , with selection for erythromycin and spectinomycin, in the presence of xylose on NA plates supplemented with 20 mM $Mg^{+2}$ and/or IPTG as required, to generate strains PDC642, PDC650, PDC651, PDC662, PDC664, PDC659, PDC678, PDC688 and PDC697.

**Two-hybrid plasmids**

*ftsE*, *ftsX* and *ftsEX* orfs were amplified by PCR from the wild-type strain 168 genomic DNA using primers ForEXbaI, RevEKpnI, ForXXbaI and RevXKpnI, and then cloned between the XbaI and KpnI sites of plasmids pUT18 and pKT25, creating the plasmids pUT18-ftsE, pUT18-FtsX, pUT18-FtsEX, pKT25-ftsE, pKT25-ftsX and pKT25-ftsEX. Corresponding plasmids pairs were used to co-transform the *E. coli* BTH101 strain for 2-hybrid analysis.

***Microscopic imaging***

For fluorescence microscopy, cells were grown to mid-exponential phase at 30°C or 37°C and mounted on microscope slides covered with a thin film of 1.2% agarose. See figure legends for specific growth conditions employed for each experiment. Fluorescence microscopy was carried out using Zeiss Axiovert 200M, Nikon Eclipse Ti-U, spinning disk confocal microscope. The images were acquired with Metamorph 6 (Molecular Devices, Inc) and FRAP-AI 7 (MAG Biosystems) software, and analyzed using ImageJ v.1.44o (National Institutes of Health). Images from a single focal plane were deconvolved using the 'No Neighbours' algorithm from the Metamorph software package. When required, cells were incubated in the presence of the membrane dye FM5-95 (90 µg $ml^{-1}$, Molecular Probes) prior to microscopic examination.

### *Sample preparation for microscopy*

For sample preparation, overnight pre-cultures of *B. subtilis* were grown in CH medium supplemented with 20 mM $MgSO_4$ (CH-Mg) and appropriate antibiotic selection, from freshly isolated colonies on plates. Day cultures were performed by diluting pre-culture to an OD600 of 0.02 in CH-Mg and grown at 30°C. Expression of fluorescent CwlO-GFP fusion was induced by addition of xylose to 0.3%. Samples for microscopic observation were taken at mid-exponential phase and immobilized on 1.2% agarose-coated microscope slides.

### *Protoplast preparation for microscopy*

Cells of strains PDC528 (wt, CwlO-$GFP_{sf}$) and PDC560 (*ΔftsX::neo*, CwlO-$GFP_{sf}$) were grown in CH media in the presence of 0.5% xylose. Cells were harvested and re-suspended in CH-MSM media in the presence of 0.5% xylose. Cells were protoplasted by incubation with 0.5 mg $ml^{-1}$ lysozyme during 30 min at 30°C. After CW removal, the protoplasts suspensions were split in two. One half was treated with proteinase K (10 µg $ml^{-1}$) for 30 min.

### *Cell measurements*

Cells from strains included in table I, constitutively expressing soluble/cytosolic GFP protein (*aprE::$P_{rpsD}$-gfp*) were grown in LB media at 37°C and samples were taken at different time points along the growth curve. Cells were imaged by epifluorescence microscopy using an Axiovert M200 microscope (Zeiss, Oberkochen, Germany) with a 300 W lambda light source (Sutter Instrument Company, California, USA) and a Zeiss x 100 plan-neofluar oil immersion objective lens (1.3 numerical aperture). Images were captured on a 1395 x 1040 pixel CoolSNAP HQ camera (Photometrics, Ottobrunn, Germany) controlled by Metamorph software version 6.1r3 (Universal Imaging Corporation, Marlow, UK). Image analysis was performed using the open source Cell Profiler software and consisted of the following two successive steps: (i) identification of cell contour by a segmentation pipeline of fluorescence images; (ii) automatic measurement of several cell characteristics: cell length and width, perimeter and area of cells. For each image, ~100-300 cells were identified and analyzed. For each strain and time point >1000 cells were analyzed.

### *Cell fractionation and immunoblotting*

The generous gift from K. Devine's laboratory of a polyclonal antibody raised against the native CwlO protein allowed us to detect it in cell fractionation experiments. In order to be able to perform pull-down experiments we constructed an epitope-tagged version of CwlO fused to the Flag tag. The CwlO-Flag fusion was expressed from the native chromosomal *cwlO* locus (strain PDC609). Flag epitope was fused to the carboxyl-terminal part of CwlO. To increase the stability of the CwlO-Flag bait, all pull down experiments were performed in a *wprA epr* double mutant background. The growth rates and cell shapes of these strains were indistinguishable from that of the wild type indicating that the fusion protein is functional.

When cells reached mid-exponential phase, cultures (50 ml) were collected by centrifugation (8,000 × g for 10 min at 25°C). Culture supernatants' protein content (S) was recovered by cold-acetone precipitation. Five volumes of cold acetone were added to 5 ml of culture

supernatant and incubated at -20°C for 1 h. Then, samples were collected by centrifugation (10,000 x g for 20 min at 4°C). Pellets were washed with 70 % cold-ethanol and air dried, before re-suspending the protein pellet in 0.5 ml of Tris buffer (100 mM Tris-HCl pH 7.5, 1x complete protease inhibitor).
Culture pellets were re-suspended in 4 mL 1× SMM buffer [0.5 M sucrose, 20 mM $MgCl_2$, 20 mM maleic acid), pH 7]; 250 µL 10 mg $ml^{-1}$ lysozyme (Sigma), and 50 µL complete protease inhibitor (EDTA-free, Roche) were added to cell suspensions and incubated at 37°C for 1 h with gentle shaking. Then cultures were split into two (2x 2 ml). First half constituted the total fraction (T). Protoplasts from the second half were collected by centrifugation. Supernatants (2 ml) were collected to constitute the CW fraction (CW). Cell membranes and cytoplasmic fractions were obtained from the protoplasts' pellets. Pellets were re-suspended in 2 ml of Tris-buffer (100 mM Tris-HCl, pH 7.5, 1x complete protease inhibitor) and sonicated until a clear solution was obtained. Membrane fraction pellets (M) were collected by centrifugation (50,000 x g for 40 min at 4°C) and supernatants were also kept as cytoplasmic fractions (C). Membrane pellets were re-suspended in 2 ml of the Tris buffer. 10 µg of total protein from each extract was separated on a 4-12% SDS-PAGE gradient gel (Novex, Life technologies). Proteins were transferred to a PVDF membrane (Amersham Hybond-P) and the membrane was blocked with 5% milk in PBST (PBS, 0.1% Tween-20) for 3 h. The membrane was incubated with appropriate antibodies (anti-Flag or anti-CwlO antibodies (1:10,000 or 1:3000 respectively in PBST)) o-n at 4°C temperature. The membrane was washed three times with PBST for 10 minutes. Following the wash, the membrane was incubated with rabbit anti-mouse or goat anti-rabbit antibodies conjugated with HRP (Sigma, A9044) (1:10,000 in 5% milk in PBST) for 1 hour at room temperature. Finally, the membrane was washed three times as above and developed using the Pierce ECL 2 Western Blotting substrate reagent. Chemiluminescence was detected using an ImageQuant LAS4000mini GE Healthcare system.

### *Formaldehyde Cross-Linking and Pull Down of CwlO Complexes*

Cross-Linking and Pull Down experiments were performed with some modifications as described by Sham *et al* 2011. Briefly, cultures (400 mL) of strains PDC612 (Bs168CA *ΔwprA::hyg Δepr::tet ΩcwlO-FLAG amyE::*$P_{xyl}$*-ftsEX-gfp*) and PDC613 (Bs168CA *ΔwprA::hyg Δepr::tet amyE::*$P_{xyl}$*-ftsEX-gfp* parent negative control) were grown exponentially to OD600 ~ 0.5. Cells were collected by centrifugation (8,000 × g for 10 min at 25°C). Cell pellets were washed with 18 mL 1× PBS at 25°C, and cells were collected again by centrifugation (8,000 × g for 5 min at 4°C). Residual supernatants were removed. Washed pellets were suspended in 19 mL 1× PBS, to which 1200 µL 37% of formaldehyde solution (Sigma) were added. Mixtures were incubated at 37 °C for 1 h. Cross-linking reactions were quenched by the addition of 4 mL 1.0 M glycine followed by incubation for 10 min at 25°C. Cells were collected by centrifugation (8,000 × g for 10 min at 4°C), washed with 20 mL 1× PBS at 25°C, and centrifuged again. Residual supernatants were removed using a fine pipette tip. Pellets of cross-linked cells were re-suspended in 5 mL 1× SMM buffer [0.5 M sucrose, 20 mM $MgCl_2$, 20 mM maleic acid), pH 7]; 250 µL 10 mg $ml^{-1}$ lysozyme (Sigma), and 50 µL complete protease inhibitor were added to cell suspensions and incubated at 37°C for 1 h with gentle shaking. Protoplast formation was monitored by phase-contrast microscopy. Protoplasts were collected by centrifugation (8,000 × g for 10 min at 4°C). Pellets were then suspended in 5 mL buffer H (20 mM Hepes, pH 8, 200 mM NaCl, 1 mM DTT, 1 x complete protease inhibitor) at 4°C. After mixing, 5 µL 0.1 M $MgCl_2$, 5 µL 0.1 M

CaCl2, 10 µL 5 mg ml$^{-1}$ DNase (D4527; Sigma), and 10 µL 10 mg ml$^{-1}$ RNase (R5500; Sigma) were added, and mixtures were incubated for 20 min on ice. Cells were then disrupted by sonication (five pulses of 40 µm amplitude for 10 s), and membrane fraction pellets were collected by centrifugation (16,000 × g for 30 min at 4°C). Membranes were dissolved in 2 mL room temperature CoIP lysis buffer [50 mM Tris·HCl, pH 7.4, 150 mM NaCl, 1 mM EDTA, 1% (vol/vol) Triton X-100], chilled, and incubated for 30 min at 4°C; 80 µL anti-FLAG M2 affinity gel (A2220; Sigma) were added. The gel was washed before use five times with 0.5 mL 1× wash buffer (50 mM Tris·HCl, pH 7.4, 150 mM NaCl, 1 mM EDTA) at room temperature as described in the manufacturer's protocol. The mixture of dissolved membranes and washed gel was added to a 2-mL gravity-flow column (Pierce) and incubated at 4°C overnight with gentle rotation. Lysate was removed, and the resin was washed three times with 1 mL CoIP lysis buffer at 25°C. FLAG-tagged protein was eluted from the column by incubation with 200 µL FLAG elution buffer (1× wash buffer containing 150 ng 3× FLAG peptide/µL) (F4799; Sigma) for 30 min at 4°C. Residual FLAG-tagged protein was eluted from the column by washing two times with 200 µL 1× wash buffer at 25°C. Eluates were filtered and concentrated (to ~40 µL) through 100-kDa cut-off Microcon columns (Millipore) by centrifugation (10,000 × g at RT). Concentrated samples were split evenly into two parts, and each one was mixed with 20 µL 2 × Laemmli sample buffer containing 5% (vol/vol) β-mercaptoethanol. One half was heated for 1 h at 95°C to remove crosslinks, while the other was kept intact. Samples were separated on 4-12% gradient SDS-PAGE gels in MES buffer and blotted into PVDF membranes, ready for immunoblotting with different antibodies (monoclonal anti-Flag and polyclonals anti-GFP, anti-Pbp2B, anti-MreB and anti-DivIVA). Finally, the membrane were developed using the Pierce ECL 2 Western Blotting substrate reagent. Chemiluminescence was detected using an ImageQuant LAS4000mini GE Healthcare system.

**Supplementary Figures**

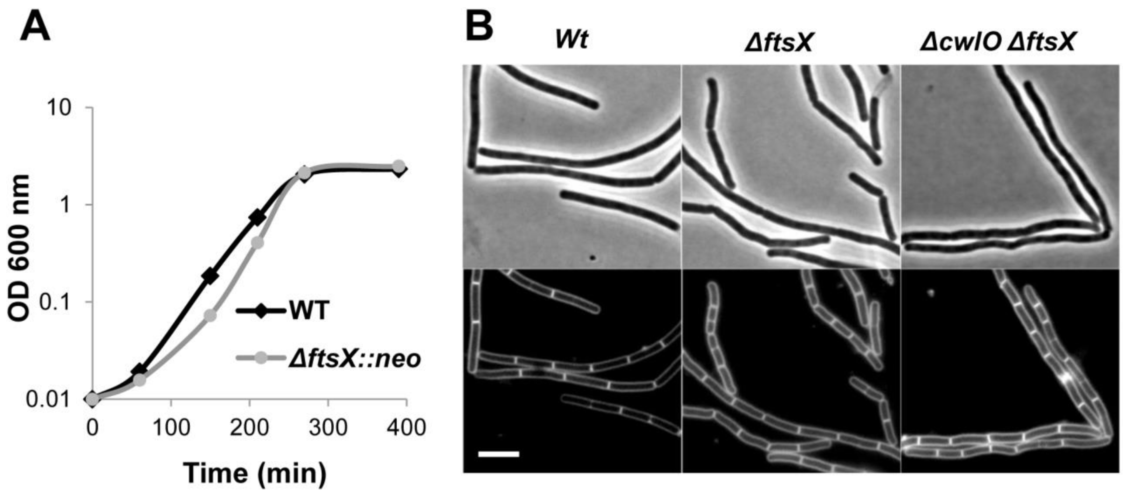


**Figure S1. Phenotypes associated with *ftsX* and *cwlO* mutant strains. (A)** Growth curve (logarithmic scale) of the wt and *fstX* null strain in liquid medium. **(B)** Cell morphologies of typical fields of strains Bs168CA, 4501 (*ΔftsX::neo*) and PDC465 (*ΔftsX::neo ΔcwlO::spec*). Scale bar represents 4 µm.

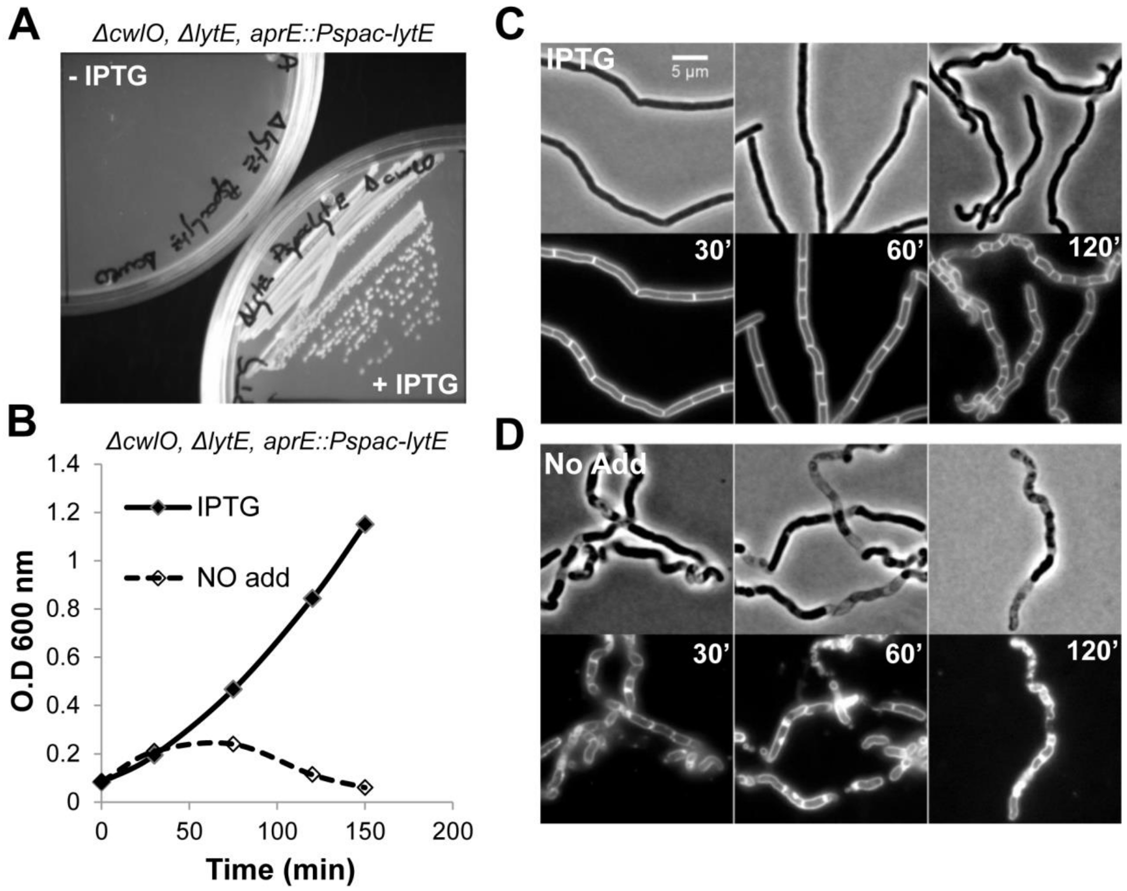


**Figure S2. LytE and CwlO mutants are synthetically lethal**. **(A)** Growth of strain PDC493 (*ΔcwlO::spec ΔlytE::cat aprE::*$P_{spac}$*-lytE)* on NA plates with or without 0.5 mM IPTG. **(B)** Growth of strain PDC493 on LB liquid medium in the presence or absence of IPTG. Growth curves (IPTG 0.5 mM, closed symbols; no addition, open symbols). **(C-D)** Effect of LytE depletion on cell morphology. Phase contrast micrographs and the corresponding membrane staining images were taken at the indicated times during the growth curves in (B). (C) 0.5 mM IPTG added, (D) No addition. The cell membranes were stained with FM5-95 membrane dye. Scale bar represents 5 μm.

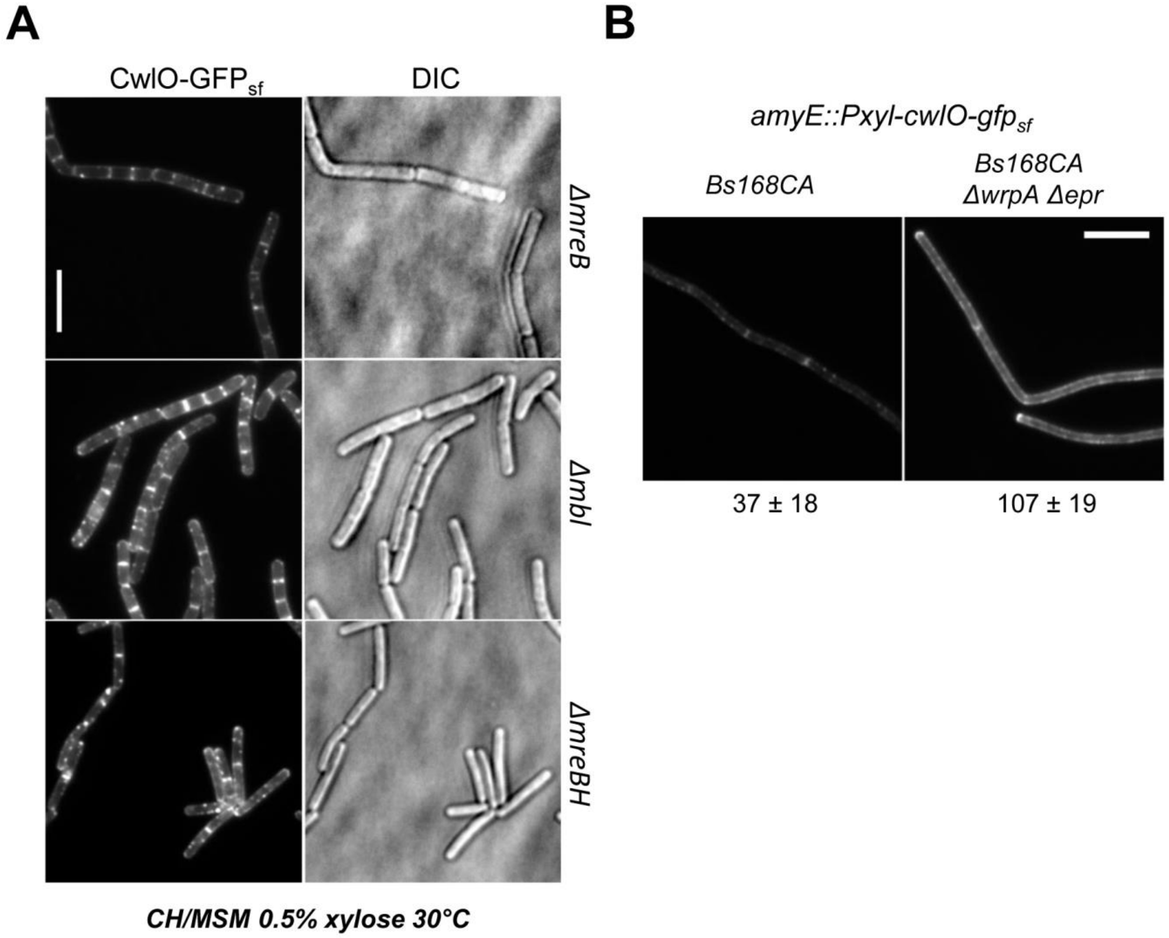


**Figure S3. CwlO localization in different genetic backgrounds.** DIC and epifluorescence microscopy of strains expressing the fluorescent fusion *amyE::P$_{xyl}$-cwlO-gfp$_{sf}$* . Cells were grown to mid-exponential phase in CH/MSM medium in the presence of 0.5% xylose at 30°C and immobilized on agarose-coated microscope slides. **(A)** The different panels correspond to strains PDC550 (*ΔmreB*), PDC552 (*Δmbl*) and PDC554 (*ΔmreBH*) as indicated. **(B)** CwlO-GFP$_{sf}$ fluorescence is increased in a surface protease-deficient background. Cells of strains PDC519 and PDC528 (*ΔwrpA Δepr)* were grown in CH media at 30°C in the presence of 0.5% xylose. Fluorescent images were taken with the same acquisition settings and exposure times with the average relative fluorescence intensity over the lateral wall of the cells indicated below. An average 2- to 3-fold increase of the brightness of the CwlO-GFP fusion in the protease-deficient relative to the wild-type background was measured in four independent experiments.

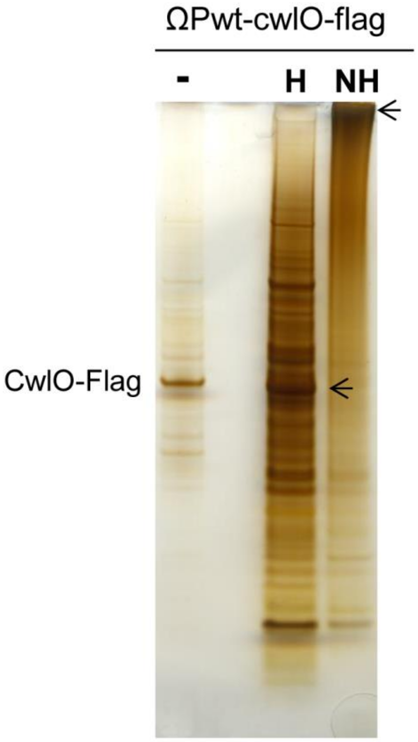


**Figure S4. FtsX and CwlO interact within the same protein complex at the cell membrane.** Pull down of cross-linked CwlO-Flag complexes in membranes detected by silver staining. Left lane, extract control sample before cross-linking. Right lanes, cross-linked samples that were heated (H) or not (NH) to break cross-links or maintain the complexes, respectively. The most prominent band in both lanes is labelled with an arrow, indicating the position of the CwlO-FLAG protein free or as part of a macro-molecular complex.

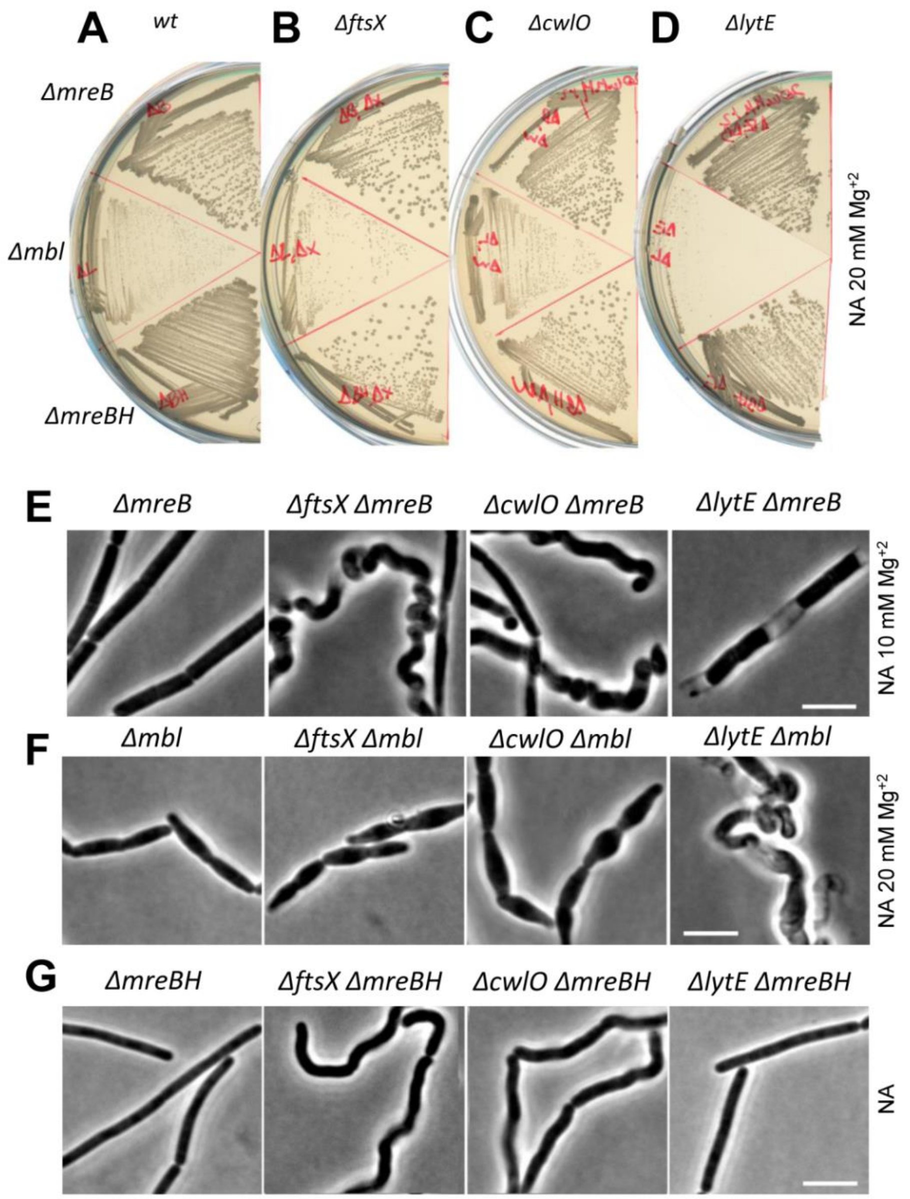


**Figure S5. LytE is synthetically lethal with Mbl (A)** Growth of strains *4281 (Ωcat 3427 ΔmreB), 4261 (Δmbl::cat) and* 4262 *(ΔmreBH::ery), and* the corresponding double mutant combinations with *ΔftsX:neo* **(B)**, *ΔcwlO::spec* **(C)** *and ΔlytE::tet* **(D)** gene deletions on NA plates in the presence of 20 mM $Mg^{+2}$. **(E)** Cell morphologies of typical fields of *mreB* mutant strain (4281) and derivatives in (B-D), growing in the presence of 10 mM of $Mg^{+2}$. Scale bar represents 5 µm. PDC454 (*ΔftsX:neo Ωcat 3427 ΔmreB*), PDC483 (*ΔcwlO::spec Ωcat 3427 ΔmreB*) and PDC577 (*ΔlytE::cat::tet ΔmreB::cat*. **(F)** Cell morphologies of typical fields of *mbl* mutant strain (4261) and derivatives in (B-D), growing in the presence of 20 mM of $Mg^{+2}$. Scale bar represents 5 µm. PDC453 (*ΔftsX:neo Δmbl*::cat), PDC473 (*ΔcwlO::spec Δmbl*::cat) and PDC576 (*ΔlytE::cat::tet Δmbl::cat*) **(G)** Cell morphologies of typical fields of *mreBH* mutant strain (4262) and derivatives in (B-D), growing on NA plates. Scale bar represents 5 µm. PDC467 *(ΔftsX:neo ΔmreBH:ery*), PDC471 (*ΔcwlO::spec ΔmreBH*:ery) *and* PDC470 *(ΔlytE::tet ΔmreBH*:ery).

## Supplementary tables

**Table S1. E-values and identity homologies identified using the basic local alignment search tool (BLAST) (Altschul *et al.*, 1990).**

| *B. subtilis* | FtsE | FtsX | CwlO | LytE |
|---|---|---|---|---|
| *E. coli* | FtsE | FtsX | EnvC | YdhO |
| | 1e-67 | 3e-22 | 2e-9 | 2.47e-19 |
| | (46%) | (28%) | (20%) | (30%) |
| *S. pneumoniae* | FtsE | FtsX | PcsB | SPD_104 |
| | 1e-111 | 1e-64 | 1e-16 | 1.5e-05 |
| | (66%) | (38%) | (30%) | (28%) |
| *B. anthracis* | FtsE | FtsX | BA5427 | BA1952 |
| | 4.85e-76 | 2.19e-98 | 2.4e-39 | 9.29e-32 |
| | (65%) | (60%) | (25%) | (29%) |

**Table S2. Bacterial strains used in this study.**

| ***B. subtilis*** **strains** | **Relevant Genotype** | **Reference** |
|---|---|---|
| Bs168CA | *trpC2* | Barbe *et al.*, 2009 |
| 1A792 | *trpC2 ΔlytABC::neo ΔlytD::tet ΔlytE::cam ΔlytF::spc* | Margot *et al.*, 1998 |
| BP079 | *trpC2 ΔcwlO::spc* | Bisicchia *et al.*, 2007 |
| WE1 | *trpC2 wprA::neo epr::tet* | Yamamoto *et al.*, 2003 |
| WB800 | *nprE aprE epr bpr mpr::ble nprB::bsr Δvpr wprA::hyg* | Wu *et al.*, 2002 |
| 2535 | Bs168ED *ΔmreBH::spc* | Carballido-Lopez *et al.*, 2006 |
| 4261 | Bs168CA *Δmbl::cat* | Schirner & Errington, 2009 |
| 4262 | Bs168CA *ΔmreBH::erm* | Schirner & Errington, 2009 |
| 4281 | Bs168CA *Ωcat 3427 ΔmreB* | Formstone & Errington, 2005 |
| 4501 | Bs168CA *ΔftsX::neo* | This study |
| 4502 | Bs168CA *ΔftsEX::neo* | This study |
| 4503 | Bs168CA *ΔftsE::neo* | This study |
| PDC453 | Bs168CA *ΔftsX::neo Δmbl::cat* | This study |
| PDC454 | Bs168CA *Ωcat 3427 ΔmreB ΔftsX::neo* | This study |
| PDC463 | Bs168CA *ΔcwlO::spc* | This study |
| PDC464 | Bs168CA *ΔlytE::cat* | This study |
| PDC465 | Bs168CA *ΔcwlO::spc ΔftsX::neo* | This study |
| PDC467 | Bs168CA *ΔmreBH::erm ΔftsX::neo* | This study |
| PDC470 | Bs168CA *ΔmreBH::erm ΔlytE::cat* | This study |
| PDC471 | Bs168CA *ΔmreBH::erm ΔcwlO::spc* | This study |
| PDC472 | Bs168CA *ΔlytE::cat aprE::*$P_{spac}$*-lytE erm* | This study |
| PDC473 | Bs168CA *ΔcwlO::spc Δmbl::cat* | This study |
| PDC478 | Bs168CA *ΔftsX::neo aprE::*$P_{spac}$*-lytE erm* | This study |
| PDC479 | Bs168CA *ΔcwlO::spc aprE::*$P_{spac}$*-lytE erm* | This study |
| PDC480 | Bs168CA *ΔftsX::neo::spc* | This study |
| PDC483 | Bs168CA *ΔcwlO::spc Ωcat 3427 ΔmreB* | This study |
| PDC484 | Bs168CA *ΔftsEX::neo::spc* | This study |
| PDC492 | Bs168CA *ΔlytE::cat ΔftsX::neo aprE::*$P_{spac}$*-lytE erm* | This study |
| PDC493 | Bs168CA *ΔlytE::cat ΔcwlO::spc aprE::*$P_{spac}$*-lytE erm* | This study |

| | | |
|---|---|---|
| PDC519 | Bs168CA *amyE::P$_{xyl}$-cwlO-gfp$_{sf}$ spc* | This study |
| PDC528 | PDC538 *amyE::P$_{xyl}$-cwlO-gfp$_{sf}$ spc* | This study |
| PDC538 | Bs168CA *wprA::hyg epr::tet* | This study |
| PDC540 | Bs168CA *aprE::PrpsD-gfp spc* | This study |
| PDC541 | Bs168CA *ΔftsX::neo aprE::PrpsD-gfp spc* | This study |
| PDC550 | PDC528 *Ωcat 3427 ΔmreB* | This study |
| PDC552 | PDC528 *Δmbl::cat* | This study |
| PDC554 | PDC528 *ΔmreBH::erm* | This study |
| PDC560 | PDC528 *ΔftsX::neo* | This study |
| PDC567 | Bs168CA *amyE::P$_{xyl}$-cwlO spc* | This study |
| PDC575 | Bs168CA *ΔlytE::cat::tet* | This study |
| PDC576 | Bs168CA *ΔlytE::cat::tet Δmbl::cat* | This study |
| PDC577 | Bs168CA *Ωcat 3427 ΔmreB ΔlytE::cat::tet* | This study |
| PDC590 | PDC567 *ΔftsX::neo* | This study |
| PDC591 | PDC567 *Ωcat 3427 ΔmreB* | This study |
| PDC592 | PDC567 *Δmbl::cat* | This study |
| PDC593 | PDC567 *ΔmreBH::erm* | This study |
| PDC594 | PDC528 *ΔftsE::neo* | This study |
| PDC609 | PDC538 *ΩPwt-cwlO-flag erm* | This study |
| PDC610 | PDC538 *ΔftsX::neo ΩPwt-cwlO-flag erm* | This study |
| PDC612 | *PDC538 ΩPwt-cwlO-flag erm amyE::P$_{xyl}$-ftsEX-gfp spc* | This study |
| PDC613 | *PDC538 amyE::P$_{xyl}$-ftsEX-gfp spc* | This study |
| PDC620 | Bs168CA *aprE::P$_{spac}$-lytE erm* | This study |
| PDC627 | Bs168CA *ΔmreBH::spc* | This study |
| PDC632 | Bs168CA *aprE::P$_{xyl}$-ftsEX erm* | This study |
| PDC639 | Bs168CA *ΔcwlO::spc aprE::P$_{xyl}$-cwlO erm* | This study |
| PDC642 | YK1119 *ΔlytE::cam::tet* | This study |
| PDC643 | Bs168 *trpC2 Δmbl ΔmreBH ΔmreB::neo amyE::P$_{spacHY}$-mreBH cat* (BH-only) | This study |
| PDC650 | YK1119 *ΔftsEX::neo::spc aprE::P$_{xyl}$-ftsEX erm* | This study |
| PDC651 | YK1119 *ΔcwlO::spc aprE::P$_{xyl}$-cwlO erm* | This study |

| | | |
|---|---|---|
| PDC659 | PDC643 *ΔcwlO::spc aprE::*$P_{xyl}$*-cwlO erm* | This study |
| PDC660 | Bs168 *trpC2 Δmbl ΔmreBH ΔmreB::neo amyE*::$P_{spacHY}$-*mreB cat* (B-only) | This study |
| PDC662 | PDC660 *ΔcwlO::spc aprE::*$P_{xyl}$*-cwlO erm* | This study |
| PDC664 | PDC660 *ΔftsEX::neo::spc aprE::*$P_{xyl}$*-ftsEX erm* | This study |
| PDC665 | PDC538 *ΔftsE::neo ΩPwt-cwlO-flag erm* | This study |
| PDC678 | YK1119 *ΔlytE::cam::tet, aprE::*$P_{xyl}$*--lytE erm* | This study |
| PDC687 | Bs168CA *ΔlytE::cam::spc* | This study |
| PDC688 | PDC660 *ΔlytE::cam::spc, aprE::*$P_{xyl}$*-lytE erm* | This study |
| PDC696 | Bs168CA *ΔlytE::cam aprE::PrpsD-gfp spc* | This study |
| PDC697 | PDC643 *ΔlytE::cam::spc, aprE::*$P_{xyl}$*-lytE erm* | This study |
| PDC702 | Bs168CA *ΔlytE::cam::spc, aprE::*$P_{xyl}$*-lytE erm* | This study |
| PDC713 | Bs168CA *Ωcat 3427 ΔmreB amyE::*$P_{xyl}$*-ftsE-gfp spc* | This study |
| PDC714 | Bs168CA *Δmbl::cat amyE::*$P_{xyl}$*-ftsE-gfp spc* | This study |
| PDC715 | Bs168CA *ΔmreBH::ery amyE::*$P_{xyl}$*-ftsE-gfp spc* | This study |
| YK1012 | Bs168 *trpC2 Δmbl ΔmreBH* | Kawai *et al.*, 2009 |
| YK1119 | Bs168 *trpC2 Δmbl ΔmreBH ΔmreB::neo amyE*::$P_{spacHY}$-*mbl cat* (BL-only) | Kawai *et al.*, 2011 |
| ***E. coli* strains** | **Relevant characteristics** | **Reference** |
| BTH101 | F- *glnV44 recA1 endA gyrA96 thi-1 hsdR17 spoT1 rfbD1 cya-854* | Karimova *et al.*, 1998 |
| DH5α | F-*endA1 hsdR17 supE44 thi-1 λ-recA1gyrA96 relA1* D(*lacZYA-argF*)*U169 φ80 dlacZ* D*M15* | GIBCO-BRL |
| XL1-Blue | *recA1 endA1 gyrA96 thi-1 hsdR17 supE44 relA1 lac* [F´ *proAB lacI*q*Z_M15* Tn*10* (Tet)] | Stratagene Ltd. |

spc, spectinomycin; kan, kanamycin; erm, erythromycin; neo, neomycin; cat, chloramphenicol; tet, tetracyclin; ble, bleomycin; bsr, blasticidin S; hyg, hygromycin. Other abbreviations: Δ, deletion; Ω, insertion.
*gfpmut1*: F64L, S65T variant of GFP (GFPmut1; (Cormack *et al.*, 1996)). $gfp_{sf}$: variant of GFP (Pedelacq *et al.*, 2006**)**

## Table S3. Plasmids used in this study.

| Plasmid | Relevant Genotype | Reference or source |
|---|---|---|
| pMUTin4 | *bla erm lacZ lacI* | Vagner *et al.*, 1998 |
| pMUTin-flag | *bla erm* $P_{spac}$-FLAG *lacI* | Kaltwasser *et al.*, 2002 |
| pAPNC213 | bla aprE::$P_{spac}$-mcs spc | Morimoto *et al.*, 2002 |
| pBEST501 | *bla neo* | Itaya *et al.*, 1989 |
| pSG1728 | bla amyE::$P_{xyl}$-mcs | Lewis & Marston, 1999 |
| pSG1154 | *bla amyE*:: $P_{xyl}$-*gfpmut1 spc* | Lewis & Marston, 1999 |
| pUT18::zip | $P_{lac}$-*zip-cyaA*$^{675\text{-}1197}$ *bla* | Karimova *et al.*, 1998 |
| pKT25::zip | $P_{lac}$-*cyaA*$^{675\text{-}1197}$-*zip bla* | Karimova *et al.*, 1998 |
| pUT18C | $P_{lac}$-*cyaA*$^{675\text{-}1197}$-mcs *bla* | Karimova *et al.*, 1998 |
| pKT25 | $P_{lac}$-*cyaA*$^{1\text{-}732}$-mcs *kan* | Karimova *et al.*, 1998 |
| pAPNC213-erm | bla aprE::$P_{spac}$-MCS erm | Olmedo-Verd, E. Unpublished |
| pUC57-gfp-sf | bla gfp-sf | Murray, H. Unpublished |
| pAPNC-PrpsD-gfp spc | pAPNC-$P_{rpsD}$-gfp spc | This study |
| pAPNC-PrpsD-gfp erm | pAPNC-$P_{rpsD}$-gfp erm | This study |
| pAPNC-erm-LytE | pAPNC-$P_{spac}$-Pwt-LytE erm | This study |
| pSG1728-cwlO | pSG1728-$P_{xyl}$-cwlO spc | This study |
| pSG1728-ftsEX | pSG1728-$P_{xyl}$-ftsEX spc | This study |
| pSG-Pxyl-cwlO-$gfp_{sf}$ | pSG1728-$P_{xyl}$-cwlO-$gfp_{sf}$ spc | This study |
| pSG1154-Pxyl-ftsEX-gfp | pSG1154-$P_{xyl}$-ftsEX-$gfp_{sf}$ spc | This study |
| pMUTin-‘cwlO-flag | pMUTin-$P_{spac}$‘cwlO-flag erm | This study |
| pAPNC-$P_{xyl}$-cwlO | pAPNC213-$P_{xyl}$-cwlO erm | This study |
| pAPNC-$P_{xyl}$-ftsEX | pAPNC213-$P_{xyl}$-ftsEX erm | This study |
| pAPNC-$P_{xyl}$-lytE | pAPNC213-$P_{xyl}$-lytE erm | This study |
| pKT25::*ftsEX* | $P_{lac}$-*cyaA*$^{1\text{-}732}$-ftsEX *kan* | This study |
| pKT25::ftsE | $P_{lac}$-*cyaA*$^{1\text{-}732}$-ftsE *kan* | This study |
| pKT25::*ftsX* | $P_{lac}$-*cyaA*$^{1\text{-}732}$-ftsX *kan* | This study |
| pUT18::*ftsE* | $P_{lac}$-ftsE-*cyaA*$^{675\text{-}1197}$ *bla* | This study |
| pUT18::*ftsX* | $P_{lac}$-ftsX-*cyaA*$^{675\text{-}1197}$ *bla* | This study |
| pUT18::*ftsEX* | $P_{lac}$-ftsEX-*cyaA*$^{675\text{-}1197}$ *bla* | This study |

MCS: Multi-cloning site, spc, spectinomycin; kan, kanamycin; erm, erythromycin; neo, neomycin; bla, ampicillin;

## Table S4. Primers used in this study

| Name | Sequence (5’-3’) |
|---|---|
| 54-GFPBamHIF | CGGGATCCGCAAACTAATGTGCAACTTAC |
| 54-gfp-BglIIrev | GCAGATCTTTTGTATAGTTCATCCATGCC |
| amyE-F1 | ACCACCAGTGATTATGCC |
| AmyEGFPsftoAprERevBamHI | CTGGATCCGCGCCGACCTTGAC |
| amyE-R1 | TGCATAACTGCTTCCAAC |
| AmyEtoAprERevBamHI | CTGGATCCCGCTCTAGAACTAGTGGATCTG |
| AprEForw | CTCTACGGAAATAGCGAGAG |
| AprERev | AGAAGCAGGTATGGAGGAAC |
| CwlOCterFHindIIIFlag | CGTAAGCTTGCTCATCTGATGATTCTTC |
| CwlO-FXhoI | CATCTCGAGCAAATGAGGACAGGTTCACAG |
| cwlORev-EcoRI | GGAATTCTTGAACAACACGTCTTACAACAC |
| CwlOrevKpnIFlag | GGGGTACCTTGTTGAACAACACGTCTTAC |
| CwlORevSacI | GATCGAGCTCTACTTGAACAACACGTCTTACAAC |
| ForA | TGAATCCCACGAAGAATTACAAATGACTCATG |
| ForB1 | ATAAAGTGAAAAAGGATCCCGTTTTCGGGACG |
| ForB2 | CAAGAGGGGAGTATGGATCCTATGATTAAAAT |
| ForEXba1 | AAGATTTCTAGATTTCATGATAGAGATGAAGGAAG |
| ForXXba1 | CAAGATCTAGATATGGTTCATATGATTAAAATTCT |
| FtsEFxhoI | CCGCTCGAGGATATAAAAGATTAGGTGATTTC |
| FtsErevEcoRI | GGAATTCATCATATGAACCATACTCCC |
| FtsEupF5 | GCTGAAGGCCAAGCTGTATC |
| FtsXdown3R | AATCTCTTTAAGCACAAAGAAACAG |
| FtsXdown5FXbaI | CATCTAGAGAAAAAGCCGTTCCGTTTTCG |
| FtsXRevEcoRI | GGAATTCTACTCGCAGAAACTTGCGG |
| GFP-sf-FEcoRI | GGAATTCACAAACATGTCAAAAGGAG |
| GFPsf-RevSpeI-NotI | CATCATAGCGGCCGCGCCGACCTTGACTAGTGCTC |
| LytEFEcoRI | GGAATTCATCGAATCTTTTCGCACCGAG |
| lytEFNheI | GATGCTAGCGTTAACATTTGGGGAGG |
| LytEF-xhoI | CGTCTCGAGGTTAACATTTGGGGAGG |
| LytEF-xmaI | TATCCCGGGGAGGAAAATATGAAAAAG |
| LytE-NcoIF | CTGACCATGGGAGTTAACATTTGGGGAG |
| LytERev-KpnI | CTGGTACCGAATCTTTTCGCACCGAGG |
| LytERevSacI | CTGAGCTCGACATCGAATCTTTTCGCACCG |
| LytERevSphI | GCACGCATGCTAGAATCTTTTCGCACCGAG |
| p+lytExhoI | CCGCTCGAGGTTATCTTGCCTTATTTGATG |
| pAPNC213-F1 | TCACTCTCAAGGCTACACAGG |
| pAPNC213-F2 | CTACAAGGTGTGGCATAATGTG |
| pAPNC213-R1 | GGTATGGAGGAACCTGCTTC |
| Pxyl-FSphI | GTGACATTTGCATGCTTCAAAG |
| RevA1 | TCACCTAATCGCATGCATCATTTTATCTATCA |
| RevA2 | TCACGCAAGTGGCATGCGAGAATTTTAATCAT |
| RevB | ACAGACACTATCTCTCACCGCCTCAAGCCAAA |
| RevEKpnI | GCGCCCGAGGTACCTAATCATATGAACCATAC |
| RevXKpnI | CGGCTTTTGGTACCTATACTCGCAGAAACTTGCGG |

## Supplementary References


Altschul, S. F., W. Gish, W. Miller, E. W. Myers & D. J. Lipman, (1990) Basic local alignment search tool. *J Mol Biol* **215**: 403-410.

Anagnostopoulos, C. & J. Spizizen, (1961) Requirements for Transformation in *Bacillus subtilis*. *J Bacteriol* **81**: 741-746.

Barbe, V., S. Cruveiller, F. Kunst, P. Lenoble, G. Meurice, A. Sekowska, D. Vallenet, T. Wang, I. Moszer, C. Medigue & A. Danchin, (2009) From a consortium sequence to a unified sequence: the *Bacillus subtilis* 168 reference genome a decade later. *Microbiology* **155**: 1758-1775.

Bisicchia, P., D. Noone, E. Lioliou, A. Howell, S. Quigley, T. Jensen, H. Jarmer & K. M. Devine, (2007) The essential YycFG two-component system controls cell wall metabolism in *Bacillus subtilis*. *Mol Microbiol* **65**: 180-200.

Carballido-Lopez, R., A. Formstone, Y. Li, S. D. Ehrlich, P. Noirot & J. Errington, (2006) Actin homolog MreBH governs cell morphogenesis by localization of the cell wall hydrolase LytE. *Dev Cell* **11**: 399-409.

Cormack, B. P., R. H. Valdivia & S. Falkow, (1996) FACS-optimized mutants of the green fluorescent protein (GFP). *Gene* **173**: 33-38.

Cutting, S. M., and P. B. Vander Horn. , (1990) Genetic analysis. In: Molecular biological methods for Bacillus. C. R. H. a. S. M. Cutting (ed). England: John Wiley & Sons Ltd. Chichester, , pp. p. 27-74.

Dinh, T. & T. G. Bernhardt, (2011) Using superfolder green fluorescent protein for periplasmic protein localization studies. *J Bacteriol* **193**: 4984-4987.

Formstone, A. & J. Errington, (2005) A magnesium-dependent mreB null mutant: implications for the role of *mreB* in *Bacillus subtilis*. *Mol Microbiol* **55**: 1646-1657.

Hamoen, L. W., W. K. Smits, A. de Jong, S. Holsappel & O. P. Kuipers, (2002) Improving the predictive value of the competence transcription factor (ComK) binding site in *Bacillus subtilis* using a genomic approach. *Nucleic Acids Res* **30**: 5517-5528.

Itaya, M., K. Kondo & T. Tanaka, (1989) A neomycin resistance gene cassette selectable in a single copy state in the *Bacillus subtilis* chromosome. *Nucleic Acids Res* **17**: 4410.

Kaltwasser, M., T. Wiegert & W. Schumann, (2002) Construction and application of epitope- and green fluorescent protein-tagging integration vectors for *Bacillus subtilis*. *Appl Environ Microbiol* **68**: 2624-2628.

Karimova, G., J. Pidoux, A. Ullmann & D. Ladant, (1998) A bacterial two-hybrid system based on a reconstituted signal transduction pathway. *Proc Natl Acad Sci U S A* **95**: 5752-5756.

Kawai, Y., K. Asai & J. Errington, (2009) Partial functional redundancy of MreB isoforms, MreB, Mbl and MreBH, in cell morphogenesis of *Bacillus subtilis*. *Mol Microbiol* **73**: 719-731.

Kawai, Y., J. Marles-Wright, R. M. Cleverley, R. Emmins, S. Ishikawa, M. Kuwano, N. Heinz, N. K. Bui, C. N. Hoyland, N. Ogasawara, R. J. Lewis, W. Vollmer, R. A. Daniel & J. Errington, (2011) A widespread family of bacterial cell wall assembly proteins. *EMBO J* **30**: 4931-4941.

Lewis, P. J. & A. L. Marston, (1999) GFP vectors for controlled expression and dual labelling of protein fusions in *Bacillus subtilis*. *Gene* **227**: 101-110.

Margot, P., M. Wahlen, A. Gholamhoseinian, P. Piggot & D. Karamata, (1998) The *lytE* gene of *Bacillus subtilis* 168 encodes a cell wall hydrolase. *J Bacteriol* **180**: 749-752.

Morimoto, T., P. C. Loh, T. Hirai, K. Asai, K. Kobayashi, S. Moriya & N. Ogasawara, (2002) Six GTP-binding proteins of the Era/Obg family are essential for cell growth in *Bacillus subtilis*. *Microbiology* **148**: 3539-3552.

Pedelacq, J. D., S. Cabantous, T. Tran, T. C. Terwilliger & G. S. Waldo, (2006) Engineering and characterization of a superfolder green fluorescent protein. *Nat Biotechnol* **24**: 79-88.

Sambrook, J., E. F. Fritsch & T. Maniatis, (1989) *Molecular Cloning: A Laboratory Manual*. Cold Spring Harbor Laboratory.

Schirner, K. & J. Errington, (2009) The cell wall regulator {sigma}I specifically suppresses the lethal phenotype of *mbl* mutants in *Bacillus subtilis*. *J Bacteriol* **191**: 1404-1413.

Vagner, V., E. Dervyn & S. D. Ehrlich, (1998) A vector for systematic gene inactivation in *Bacillus subtilis*. *Microbiology* **144 ( Pt 11)**: 3097-3104.

Wu, S. C., J. C. Yeung, Y. Duan, R. Ye, S. J. Szarka, H. R. Habibi & S. L. Wong, (2002) Functional production and characterization of a fibrin-specific single-chain antibody fragment from *Bacillus subtilis*: effects of molecular chaperones and a wall-bound protease on antibody fragment production. *Appl Environ Microbiol* **68**: 3261-3269.

Yamamoto, H., S. Kurosawa & J. Sekiguchi, (2003) Localization of the vegetative cell wall hydrolases LytC, LytE, and LytF on the *Bacillus subtilis* cell surface and stability of these enzymes to cell wall-bound or extracellular proteases. *J Bacteriol* **185**: 6666-6677.
